# Chromatographic separation of glycated peptide isomers derived from glucose and fructose

**DOI:** 10.1007/s00216-022-04243-9

**Published:** 2022-08-03

**Authors:** Sebastian Schmutzler, Ralf Hoffmann

**Affiliations:** 1 Institute of Bioanalytical Chemistry, Faculty of Chemistry and Mineralogy, Universität Leipzig, Leipzig, Germany; 2 Center for Biotechnology and Biomedicine, Universität Leipzig, Leipzig, Germany; 3 Institut für Bioanalytische Chemie, Biotechnologisch-Biomedizinisches Zentrum, Deutscher Platz 5, 04103 Leipzig, Germany

**Keywords:** Amadori and Heyns peptides, Fructation, Glucation, Hydrophilic interaction chromatography (HILIC), Reversed-phase high-performance liquid chromatography (RP-HPLC)

## Abstract

Amino groups in proteins can react with aldehyde groups in aldoses or keto groups in ketoses, e.g., d-glucose and d-fructose, yielding Schiff bases that rearrange to more stable Amadori and Heyns products, respectively. Analytical strategies to identify and quantify each glycation product in the presence of the corresponding isomer are challenged by similar physicochemical properties, impeding chromatographic separations, and by identical masses including very similar fragmentation patterns in tandem mass spectrometry. Thus, we studied the separation of seven peptide families, each consisting of unmodified, glucated, and fructated 15mer to 22mer peptides using reversed-phase (RP) and hydrophilic interaction chromatography (HILIC). In RP-HPLC using acidic acetonitrile gradients, unglycated peptides eluted ~ 0.1 to 0.8 min after the corresponding glycated peptides with four of seven peptides being baseline separated. Isomeric glucated and fructated peptides typically coeluted, although two late-eluting peptides were partially separated. Neutral eluents (pH 7.2) improved the chromatographic resolution (*R*_s_), especially in the presence of phosphate, providing good and often even baseline separations for six of the seven isomeric glycated peptide pairs with fructated peptides eluting earlier (*R*_s_ = 0.7 to 1.5). Some glucated and unmodified peptides coeluted, but they can be distinguished by mass spectrometry. HILIC separated glycated and unmodified peptides well, whereas glucated and fructated peptides typically coeluted. In conclusion, HILIC efficiently separated unmodified and the corresponding glycated peptides, while isomeric Amadori and Heyns peptides were best separated by RP-HPLC using phosphate buffered eluents.

**Supplementary Information:**

The online version contains supplementary material available at 10.1007/s00216-022-04243-9.

## Introduction

The Maillard reaction starts with a non-enzymatic reaction of carbonyl groups in reducing sugars, including d-glucose and d-fructose, and amino groups yielding initially Amadori (ARP) and Heyns rearrangement products (HRP), respectively, and after consecutive reactions complex mixtures of advanced glycation end products (AGEs). As the intake of fructose-containing products has substantially increased, e.g., soft drinks rich in high-fructose corn syrup and juices, both fructose metabolism and its contribution to glycation in vivo have to be further investigated. Fructose is ubiquitously added to processed food as an intense sweetener, which appears to significantly contribute to the growing numbers of non-communicable diseases (NCDs), including cardiovascular disease, fatty liver disease, and type 2 diabetes [1]. Considering the high glucose concentrations in blood, cells, and tissues, glucation of lysine and arginine residues in proteins has been extensively studied as part of regular aging and pathological processes, especially diabetes, including the chemical and structural features of individual sites [2]. Hyperglycemic conditions trigger extensive protein glycation affecting transport and immune functions and leading over the course of many years to extensive accumulations of AGEs, which are evidently correlated with the pathogenesis of diabetic complications and rheumatoid arthritis [3]. Specific glycation sites of human serum albumin and haptoglobin may indicate fluctuating blood glucose levels more sensitive than N-terminally glycated hemoglobin A (i.e., HbA_1c_), and thus might be of additional diagnostic relevance [4]. Despite many studies focusing on fructation in vitro, the importance of fructose-derived Maillard products in vivo, especially about the site specificity, remains unclear despite a pivotal role of fructose in intestines and organs using the polyol pathway [5]. Nevertheless, fructose and fructation products appear to contribute more to glycoxidation than glucose favoring the formation of harmful AGEs [6]. The complexity of even simple model systems was indicated for a mixture of xylose and glycine, where more than one hundred different products were identified using thin-layer (TLC) and high-performance liquid chromatography (HPLC) [7]. Reversed-phase (RP-) HPLC provides a high resolution for peptides and can be coupled on-line to electrospray ionization mass spectrometry (ESI–MS) enabling bottom-up proteomics. However, RP-HPLC does not well separate Amadori and Heyns peptides, as the polar hexosamine modification does not contribute much to retention, which is mainly driven by hydrophobic interactions [8]. Consequently, it is difficult to separate glycated and the corresponding unmodified peptides and even more challenging to separate isomeric glycated peptides, such as hexose-derived Amadori and Heyns peptides. This is also true for eluent systems containing trifluoroacetic (TFA) or formic acid (FA) and acetonitrile or methanol [9], while the more hydrophobic ion-pair reagent heptafluorobutyric acid (HFBA) partially provides a better separation [9]. However, HFBA is unfavorable for ESI–MS. Recently, nonafluoropentanoic acid has been frequently used for the separation of small unglycated and glycated molecules [10, 11], but studies on peptides are missing. While stronger ion-pair reagents are advantageous for the separation of unmodified and glycated peptides, they do not improve the separation of glucose- and fructose-derived peptide isomers, even for pentapeptides and shallow gradients [12]. Coeluting glucated and fructated amino acids or peptides can be analyzed by multiple reaction monitoring (MRM) in ESI–MS using isomer-typical transitions [11, 13, 14]. However, these transitions are unspecific and misleading for many sequences [11, 14], which necessitates at least a partial chromatographic separation.

Hydrophilic interaction chromatography (HILIC) has been successfully applied to analyze polar compounds, including carbohydrates, amino acids, small peptides, and O- or N-glycopeptides [15–18]. Hence, HILIC appears to be promising for the separation of glycated peptides, especially as it can be coupled on-line to ESI–MS. Depending on the stationary and mobile phases, HILIC can be considered as a partition chromatography with an aqueous layer on the polar stationary phase, where analytes can additionally adsorb by polar or ionic interactions [16, 19]. As retention relies mostly on polar interactions, HILIC has been successfully used for glycated amino acids and peptides up to four residues [20–22], including tetrapeptides glycated by different sugars at the ɛ-amino groups of lysine residues [21].

As comprehensive studies on the separation of longer unmodified, glucated, and fructated peptides are missing, this study evaluates the separation of unmodified, glucated, and fructated peptides for seven different sequences using acidic and neutral eluents in RP-HPLC on C_18_-bonded silica phases and HILIC on a crosslinked diol stationary phase. A neutral aqueous acetonitrile gradient containing phosphate buffer allowed a good separation of glucated and fructated peptides in RP-HPLC, while HILIC mostly provided baseline separations for homologous unmodified and glycated peptides.

## Materials and methods

### Reagents and materials

Reagents were obtained from the following companies: Biosolve B.V. (Valkenswaard, Netherlands): acetonitrile (ULC-MS grade, > 99.97%) and formic acid (FA, > 99%, ULC-MS grade); Fluka Analytical (Seelze, Germany): ammonium acetate (≥ 99.0%, LC–MS grade); Sigma-Aldrich (Steinheim, Germany): ammonium formate (≥ 99.0%), formic acid (FA, ~ 98%, LC–MS grade), potassium phosphate dibasic (≥ 99.0%, anhydrous), potassium phosphate monobasic (≥ 99.5%, anhydrous), sodium phosphate dibasic dodecahydrate (≥ 99.0%), sodium phosphate monobasic dihydrate (≥ 99.0%), and TFA (≥ 99%, HPLC grade); and VWR International GmbH (Darmstadt, Germany): acetonitrile (≥ 99.9%).

Water was purified in-house (resistance ≥ 18 mΩ, total organic content < 1 ppb) using a PureLab Ultra Analytic System (ELGA Lab Water, Celle, Germany). All columns were equipped with Security Guard columns from Phenomenex Ltd. (Aschaffenburg, Germany). Amadori, Heyns, and the corresponding unmodified peptides were synthesized on solid phase, as previously described [4, 14, 23]. Briefly, peptides were synthesized employing Fmoc/^*t*^Bu chemistry and DIC/HOBt activation on Fmoc-l-Lys(Boc)- or Fmoc-l-Arg(Pbf)-Wang resins. Fructated lysine residues were incorporated as Fmoc-Lys(Glc/Man,Boc)-OH. Lysine residues to be glucated were selectively deprotected and the peptide incubated with glucose in DMF at 110 °C. Peptides were cleaved with TFA containing a scavenger mixture (12.5%, v/v; 1,2-ethandithiole, *m*-cresol, thioanisole, and water; 1/2/2/2, v/v/v/v) for 2 h and precipitated with diethyl ether. Cysteine-containing peptides were reduced with TCEP and carbamidomethylated with iodoacetamide. Peptides were purified by RP-HPLC using acetonitrile gradients in the presence of 0.1% TFA and reconstituted in 20% (v/v) aqueous acetonitrile (1.5 mmol/L).

### IP-RP-HPLC

Separations used a Jupiter C_18_ column (ID: 2 mm, length: 150 mm, particle size: 5 µm, pore size: 300 Å, Phenomenex) and a System Gold HPLC equipped with a 508 autosampler (100 µL injection volume), a 125NM binary gradient pump, and a UV detector (Knauer GmbH, Berlin, Germany). Eluents were 0.1% (v/v) TFA (eluent A1) and 60% (v/v) aqueous acetonitrile containing 0.1% (v/v) TFA (eluent A2). Peptides were eluted using a linear acetonitrile gradient from either 5 to 95% eluent A2 in 30 min or 5 to 60% eluent A2 in 55 min. Separations were performed at a column temperature of 60 °C with a flow rate of 0.2 mL/min, and the absorbance was recorded at 214 nm. Separations were performed twice with deviations of the retention times of ~ 0.2 min for the shallow gradient.

### RP-HPLC at neutral pH

Separations used either a Synergi Fusion-RP C_18_ column (ID: 2 mm, length: 150 mm, particle size: 4 µm, pore size: 80 Å, Phenomenex) or Aqua C_18_ column (ID: 2 mm, length: 150 mm, particle size: 3 µm, pore size: 125 Å, Phenomenex) using a System Gold HPLC equipped with a 507e autosampler (100 µL injection volume), a 125NM binary gradient pump, and a 166 UV/VIS detector. Eluents were water (eluent B1) and aqueous acetonitrile (60% v/v, eluent B2 or 40% v/v, eluent B3) containing sodium or potassium phosphate buffer or ammonium acetate (10 mmol/L). Eluents were prepared from aqueous stock solutions (0.1 mol/L) adjusted to pH 7.2, in case of ammonium acetate with ammonia. Solvents were filtered (pore size, 0.2 µm; Pall Corp., Ann Arbor, MI) and sonicated for 15 min prior to use. Peptides were eluted using a linear acetonitrile gradient from either 5 to 95% eluent B2 in 30 min, 5 to 70% eluent B2 in 65 min, or 7.5 to 95% eluent B3 in 58 min. Unless otherwise indicated, separations were performed at 60 °C with a flow rate of 0.2 mL/min and the absorbance was recorded at 214 nm. Fractions were analyzed by matrix-assisted laser desorption/ionization time-of-flight MS (MALDI-TOF/TOF–MS) on a 5800 proteomic analyzer (ABSciex GmbH, Darmstadt, Germany) operating in reflector mode and using a solution of α-cyano-4-hydroxycinnamic acid (4 g/L) in eluent A2 as matrix.

#### RP-HPLC-ESI-IT-MS

Separations used a Jupiter C_18_ column (see “IP-RP-HPLC”) and a 1100 LC system (Agilent Technologies, Santa Clara, CA, USA) equipped with an UV detector coupled on-line to an ion trap mass spectrometer equipped with an electrospray ionization source (ESI-IT-MS, Esquire HCT, Bruker Daltonics) operated in positive ion mode. Eluents were formic acid (0.1% v/v; eluent C1) and aqueous acetonitrile (60% v/v) containing formic acid (0.1% v/v; eluent C2). Gradients used a linear slope from either 5 to 95% eluent C2 in 30 min or 5 to 60% eluent C2 in 55 min. The column temperature was 60 °C, the flow rate 0.2 mL/min, and the injection volume 100 µL. The absorbance was recorded at 214 nm. The ESI source was operated at a source temperature of 365 °C using nitrogen as curtain gas (40 psi) and dry gas (9 L/min).

### nanoUPLC-ESI-Orbitrap-MS/MS

Peptides were analyzed on a nanoAcquity UPLC (Waters GmbH) coupled on-line to an LTQ Orbitrap XL ETD mass spectrometer equipped with a nano-ESI source (Thermo Fisher Scientific GmbH, Bremen, Germany) operated in positive ion mode. Eluents were aqueous formic acid (0.1% v/v; eluent D1) and acetonitrile-containing formic acid (0.1% v/v; eluent D2). Peptides (10 µL, 500 fmol each) were trapped on a nanoAcquity Symmetry C_18_ column (ID: 180 μm, length: 2 cm, particle diameter: 5 μm) at a flow rate of 5 μL/min (3% eluent D2). Separation was achieved on a BEH 130 column (C_18_ phase, ID: 75 μm, length: 10 cm, particle diameter: 1.7 μm) using a flow rate of 0.4 μL/min and a column temperature of 30 °C. Peptides were eluted with two linear gradients from 3 to 40% eluent D2 in 87 min and then to 85% eluent D2 in 5 min. The transfer capillary temperature was set to 200 °C, and an ion spray voltage of 1.4 kV was applied to a PicoTip™ on-line nano-ESI emitter (New Objective, Berlin, Germany). Mass spectra (*m/z* range 400 to 2000) were recorded in the Orbitrap mass analyzer at a resolution of 60,000 at *m/z* 400. Tandem mass spectra were acquired in CID mode (isolation width 2 m*/z* units, normalized collision energy 35%, activation time 30 ms, default charge state 2, intensity threshold of 500 counts) using data-dependent acquisition (DDA) for the six most intense signals with a dynamic exclusion window of 60 s.

### HILIC

Peptides were separated by HILIC using a Luna HILIC column (ID: 2 mm, length: 100 mm, particle size: 3 µm, pore size: 200 Å, Phenomenex) and a System Gold HPLC equipped with a 507e autosampler (20 µL injection volume, full loop injection), a 125NM binary gradient pump, and a 166 UV–VIS detector. Eluents were 90% (v/v) aqueous acetonitrile (eluent E1) and 50% (v/v) aqueous acetonitrile (eluent E2), both containing ammonium formate (5 mmol/L). Eluents were prepared from an aqueous ammonium formate stock solution adjusted with formic acid to pH 3.2. Solvents were filtered (pore size, 0.2 µm; Pall Corp., Ann Arbor, MI) and sonicated for 15 min prior to use. Peptide standards (1.5 mmol/L in 20% (v/v) aqueous acetonitrile) were pre-diluted tenfold in 20% (v/v) aqueous acetonitrile before eluent E1 was added in two portions to achieve a sixfold dilution (25 µmol/L in 78% (v/v) aqueous acetonitrile containing 4.2 mmol/L ammonium formate) with brief vortexing and centrifugation after each dilution step. Peptides were eluted by a linear water gradient from 5 to 95% eluent E2 in 20 min or 60 min triggered 5 min after sample injection. Separations were performed at room temperature using a flow rate of 0.2 mL/min. The absorbance was recorded at 214 nm.

### Data analysis

Peak width at half height (*w*_h_) and peak asymmetry, calculated by the tailing factor (*T*_10%_), were determined for signals in UV chromatograms using the Beckman Coulter 32 Karat v5.0 software package, while Skyline (20.2.0.343, MacCoss Lab, Department of Genome Sciences, University of Washington) was used to calculate *w*_h_ in extracted ion chromatograms (XICs). Assuming Gaussian peaks, *w*_h_ was used to calculate the peak width at the base (*w*_b_) and the chromatographic resolution (*R*_s_).

Physicochemical properties relevant for chromatographic separation, i.e., isoelectric points (pI), partition coefficients (log*P*), and distribution coefficients (log*D*), were predicted using ChemAxon Instant JChem software (version 18.28.0, ChemAxon, Budapest, Hungary, www.chemaxon.com).

## Results and discussion

As isomeric glucated and fructated peptides cannot be differentiated by mass spectrometry, they have to be separated first be liquid chromatography, which is already challenging for medium-sized peptides due to minor structural differences in the polar sugar moiety. Here, we focused on RP-HPLC and HILIC, as standard methods for analyzing peptides by on-line ESI–MS. The separation efficiency was tested for seven peptide sequences previously identified in tryptic digests of human plasma as promising biomarkers for type 2 diabetes [4, 24, 25], with each sequence synthesized as unmodified (except peptide #1), glucated, and fructated peptide (Table 1).

**Table 1:** Synthetic peptides derived from tryptic sequences representing specific glycation sites in plasma proteins

#	Protein/location		Sequence	pI	Log*P*	Log*D* (pH 2.2)	Log*D* (pH 3.2)	Log*D* (pH 7.4)
1	HP A_77_-K_93_ **K**_81_	B	AVGD**K**_Ama_LPEC*EAVC*GKPK	6.18	− 21.9	− 24.7	− 23.4	− 22.0
		C	AVGD**K**_Hey_LPEC*EAVC*GKPK	6.15	− 22.1	− 24.9	− 23.6	− 22.2
2	HSA A_258_-K_274_ **K**_262_	A	ADLAKYIC*ENQDSISSK	4.34	− 19.9	− 21.5	− 20.3	− 22.7
		B	ADLA**K**_Ama_YIC*ENQDSISSK	4.34	− 21.9	− 23.5	− 22.4	− 24.7
		C	ADLA**K**_Hey_YIC*ENQDSISSK	4.34	− 22.0	− 23.7	− 22.5	− 24.9
3	HSA T_52_-K_73_ **K**_64_	A	TC*VADESAENC*DKSLHTLFGDK	4.01	− 23.2	− 23.8	− 23.2	− 36.0
		B	TC*VADESAENC*D**K**_Ama_SLHTLFGDK	3.95	− 25.2	− 25.8	− 25.2	− 38.0
		C	TC*VADESAENC*D**K**_Hey_SLHTLFGDK	3.95	− 25.4	− 26.0	− 25.4	− 38.2
4	HSA K_414_-K_428_ **K**_414_	A	KVPQVSTPTLVEVSR	9.11	− 12.6	− 16.9	− 16.3	− 13.4
		B	**K**_Ama_VPQVSTPTLVEVSR	8.35	− 14.9	− 19.0	− 18.3	− 15.3
		C	**K**_Hey_VPQVSTPTLVEVSR	8.21	− 15.0	− 19.1	− 18.5	− 15.3
5	HSA V_373_-K_389_ **K**_378_	A	VFDEFKPLVEEPQNLIK	3.99	− 11.5	− 13.0	− 11.8	− 17.3
		B	VFDEF**K**_Ama_PLVEEPQNLIK	3.99	− 13.6	− 15.0	− 13.8	− 19.4
		C	VFDEF**K**_Hey_PLVEEPQNLIK	3.99	− 13.7	− 15.2	− 14.0	− 19.6
6	HSA A_226_-K_240_ **K**_233_	A	AEFAEVSKLVTDLTK	4.43	− 13.2	− 14.9	− 13.9	− 15.9
		B	AEFAEVS**K**_Ama_LVTDLTK	4.43	− 15.2	− 16.9	− 15.9	− 18.0
		C	AEFAEVS**K**_Hey_LVTDLTK	4.43	− 15.4	− 17.1	− 16.0	− 18.1
7	HSA E_542_-K_557_ **K**_545_	A	EQLKAVMDDFAAFVEK	4.01	− 12.3	− 13.7	− 12.5	− 18.2
		B	EQL**K**_Ama_AVMDDFAAFVEK	4.01	− 14.4	− 15.7	− 14.5	− 20.2
		C	EQL**K**_Hey_AVMDDFAAFVEK	4.01	− 14.5	− 15.9	− 14.7	− 20.4

*Ama* fructosamine-modified lysine, *Hey* gluco-/mannosamine modified lysine

^*^Carbamidomethylation with iodoacetamide

### IP-RP-HPLC

**Fig. 1:**
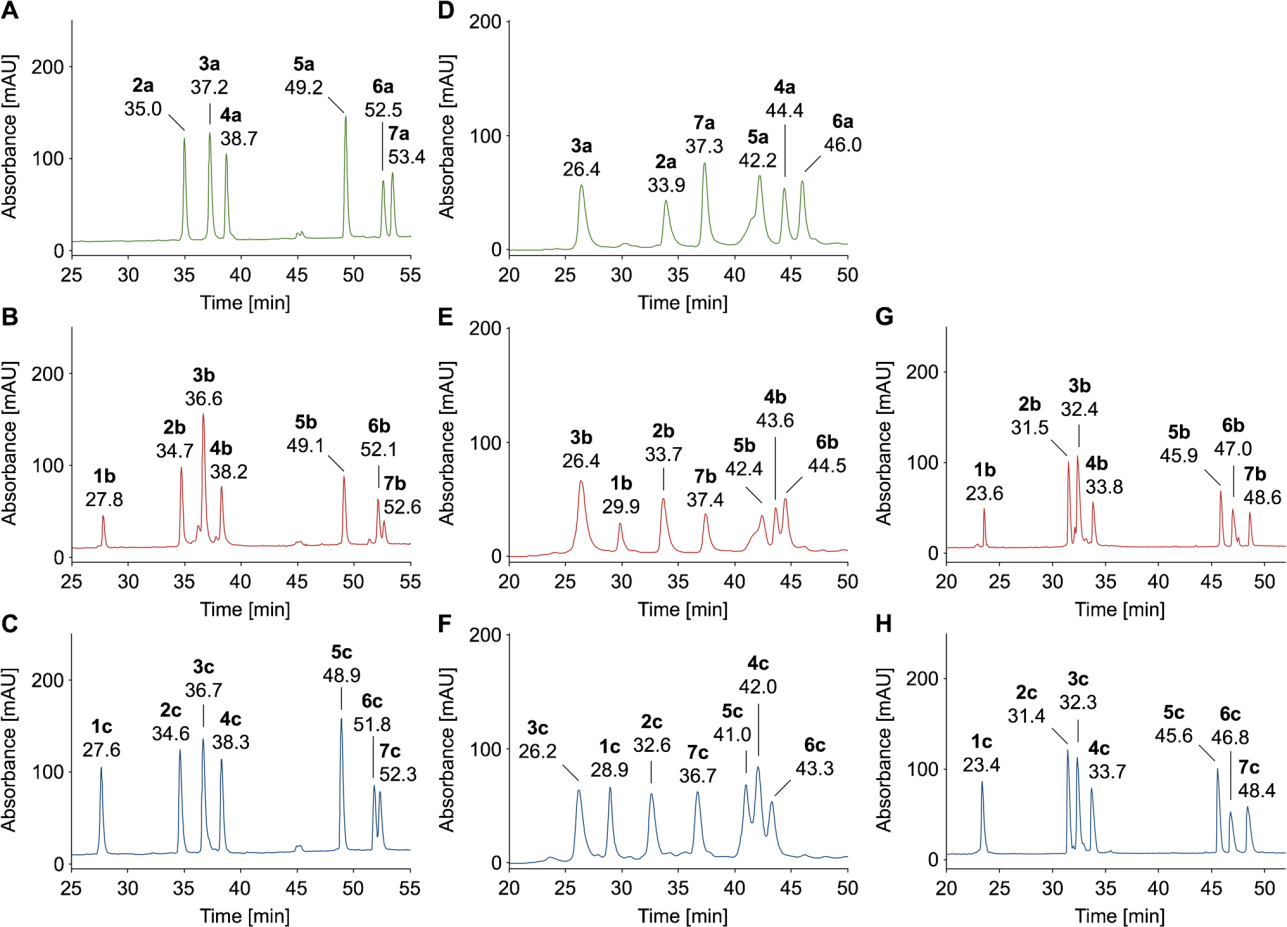
RP chromatograms of unmodified (green traces, *n* = 6), glucated (red traces, *n* = 7), and fructated (blue traces, *n* = 7) peptides (500 pmol each) showing the part of the chromatogram where the peptides eluted. Peptides were separated on a Jupiter C_18_ column (**A**–**C**, **G**–**H**) or Synergi Fusion RP column (**D**–**F**) at 60 °C using eluent systems A (**A**–**C**), B (**D**–**F**), or C (**G**–**H**) and gradients with a slope of 0.6% acetonitrile per minute. Absorbance was recorded at 214 nm. Full chromatograms are provided in the Supplement (Fig. S2, S12, S14). Peptide sequences and modification sites are provided in Table 1

The standard eluent system for peptide separation in RP-HPLC, i.e., aqueous acetonitrile containing 0.1% TFA, eluted the six unmodified and fourteen glycated peptides at acetonitrile contents of ~ 13% (#1c; 27.6 min) to ~ 28% (#7a; 53.4 min) on a Jupiter C_18_ column (Fig. 1A–C; Tab. S1). All unmodified peptides were separated, four with baseline separation and peptides #6a and #7a with a resolution of ~ 1.9. The glycated peptides eluted ~ 0.1 to 0.8 min earlier, but showed a very similar elution profile with five peptides baseline separated and peptides #6 and #7 again partially separated (*R*_s_ = 1.1). All peptides eluted as sharp symmetrical peaks (*w*_h_ < 0.3 min, *T*_10%_ < 1.5), but the retention times of glucated and fructated peptide isomers were very similar and they coeluted in broad peaks with the unmodified peptides when injected as a mixture (Fig. S3). Peptides could be grouped by retention times in early (#1), mid (#2, #3, #4), and late (#5, #6, #7) eluting peptides. The distribution coefficients (log*D*) calculated for pH 2.2 (Table 1) [26, 27] correlated better with retention times (Spearman rank correlation coefficients *r*_S_ = 0.83) than the partition coefficients (log*P*), but both did not predict the elution order well. The best separation was achieved for late-eluting modified and unmodified peptides (#5: *R*_s_ = 0.8, Fig. S3E), especially for partially resolved fructated, glucated, and unmodified peptides #6 eluting at 51.9 min, 52.2 min, and 52.5 min, respectively (Fig. S3F). The coelution of most glycated and unmodified peptides of a given sequence can be explained by the minor influence of the polar sugar moieties on peptide retention in RP-HPLC. Glycation appears to affect only retention of hydrophobic peptides, as the polar sugar may disturb strong extended hydrophobic interactions including a diffusion of the peptide into the C_18_ bonded phase. Acids as ion-pair reagents improve retention via interaction with the positively charged lysine residues. Glycated lysine residue is still able to form such an ion pair, but the nearby sugar moiety will most likely disturb the interaction between the ion pair and the C_18_ phase, which explains also the earlier elution of glycated peptides compared to unmodified peptides.

### RP-HPLC at neutral pH

**Fig. 2:**
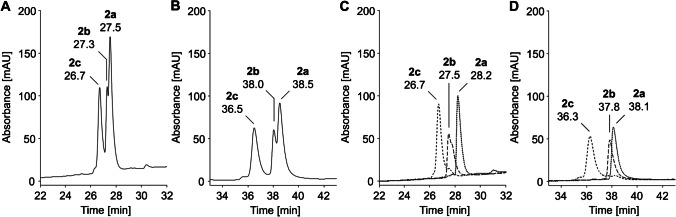
RP chromatograms of unmodified (8**a**), glucated (8**b**), and fructated (8**c**) peptides (500 pmol each) of peptide family #2 (panels A and B) and an overlay of the RP chromatograms of individually injected peptides (panels C and D) displayed from 22 to 32 min and 33 to 43 min, respectively. Separations were performed on an Aqua C_18_ column at a column temperature of 60 °C using eluent system B and gradients with a slope of 1.8% (panels A and C) or 0.6% acetonitrile per minute (panels B and D). Absorbance was recorded at 214 nm. The full chromatograms are provided in the Supplement (Fig. S5, S6). Peptide sequences and modification sites are provided in Table 1

A previous report indicated that RP-HPLC using phosphate-buffered methanol eluents might be able to separate glucated and fructated dipeptides, although this was not confirmed for a mixture of both isomeric peptides [28]. Thus, we replaced TFA in eluent system A by sodium phosphate (10 mmol/L, pH 7.2; eluent system B). The higher pH reduces and inverts the charge of the peptides, for example, for peptide #3 from + 3.8 to − 3.0 (Tab. S2), which will have a major effect on the peptide-C_18_ interactions and thus alter peptide retention. Ionic interactions with silica should be mostly suppressed for polar-endcapped (Aqua) and polar-embedded (Synergi Fusion RP) C_18_ phases. These stationary phases supposedly provide a better selectivity for polar peptides by hydrogen bonds [29], which should also enhance interactions with the sugar moieties in glycated peptides. Peptide family #2, which coeluted on a Jupiter C_18_ column using eluent system A (0.1% TFA), was partially separated on an Aqua C_18_ column using a fast gradient (slope of 1.8% acetonitrile per min) of eluent system B. A threefold shallower gradient provided almost a baseline separation (*R*_s_ = 1.6) of the first two analytes (*t*_r_ = 36.5 and 38.0 min) and a partial separation (*R*_s_ = 0.6) of the third analyte eluting at 38.5 min (Fig. 2A and B). Individual injections of the peptides revealed that fructated peptide #2c eluted first followed by glucated peptide #2b and unmodified peptide #2a (Fig. 2C and D). The good resolution was limited by peak broadening (*w*_h_ = 0.47–0.64 min) and peak tailing (*T*_10%_ = 1.63–2.25). Undesirably, the column backpressure gradually increased despite extended equilibration phases and a stability of the silica-based guard and analytical columns up to pH 7.5, most likely due to the high column temperature of 60 °C applied at pH 7.2.

**Fig. 3:**
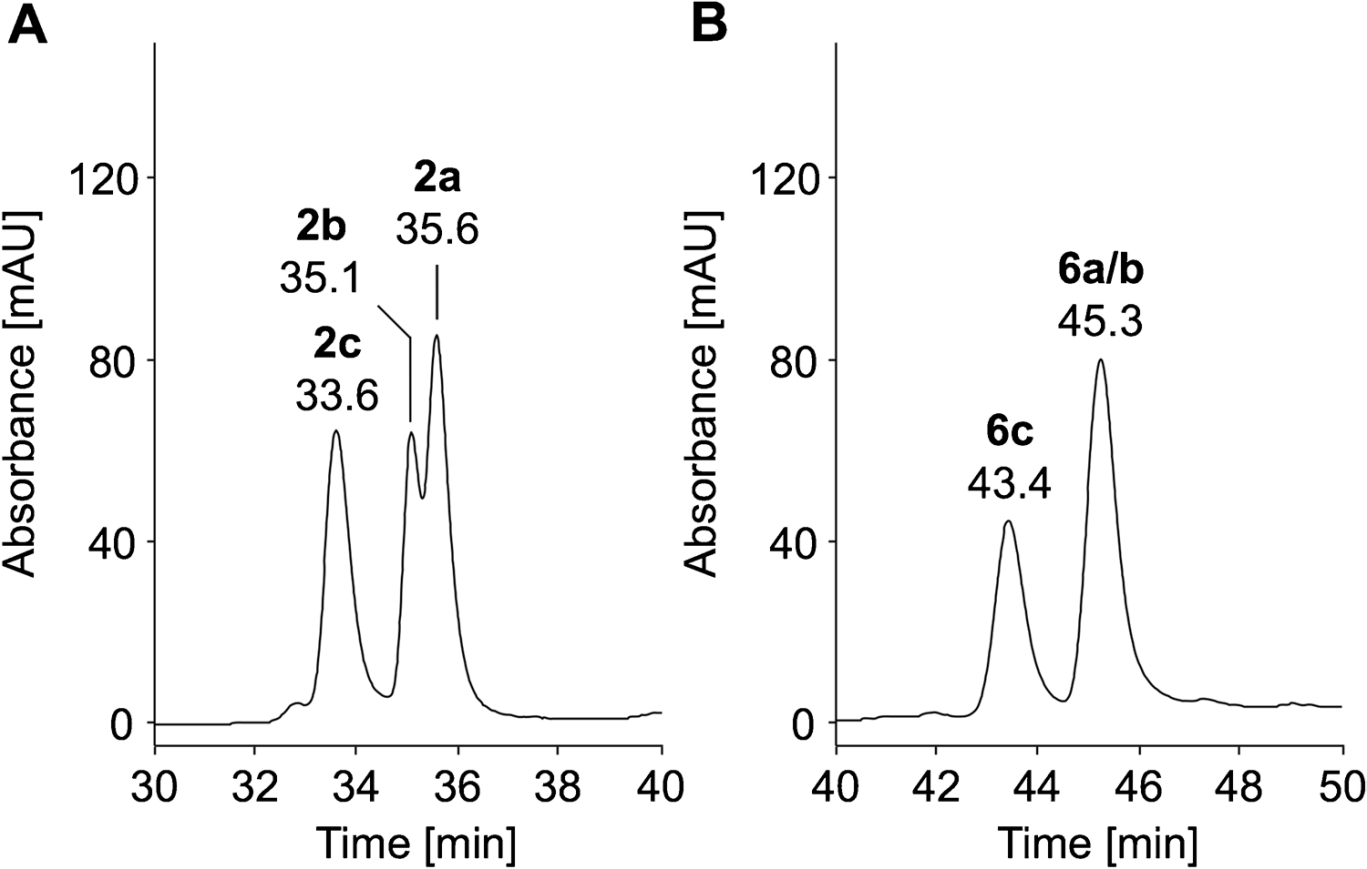
RP chromatograms of unmodified (**a**), glucated (**b**), and fructated (**c**) peptides (500 pmol each) of peptide family #2 (panel A) and #6 (panel B) displayed from 30 to 40 min and 40 to 50 min, respectively. Peptides were separated on a Synergi Fusion RP column (60 °C) using a linear 65-min gradient from 3 to 42% aqueous acetonitrile containing sodium phosphate (10 mmol/L, pH 7.2). Absorbance was recorded at 214 nm. The full chromatograms are provided in the Supplement (Fig. S7). Peptide sequences and modification sites are provided in Table 1

**Fig. 4:**
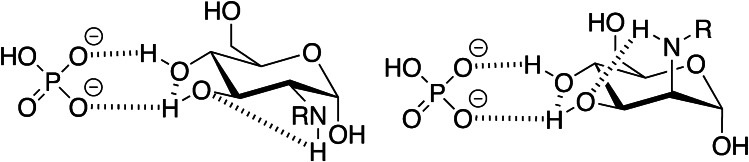
Possible hydrogen bonding interactions of 2-amino-2-deoxyglucosyl- and 2-amino-2-deoxymannosyllysine modification (HRP) with hydrogen phosphate

A Synergi Fusion RP column with a polar-embedded C_18_ phase stable up to pH 8 at 60 °C allowed an equally efficient separation (*R*_s_ = 1.5/0.5) of peptide family #2 (Fig. 3A) including peak shapes, but all peptides eluted ~ 2.9 min earlier. The chromatogram of peptide family #6 displayed only two baseline separated peaks (*R*_s_ = 1.7) with the first peak (*t*_r_ = 43.4 min, *w*_h_ = 0.67 min) corresponding to fructated peptide #6c and the second peak (*t*_r_ = 45.3 min, *w*_h_ = 0.66 min) representing coeluting unmodified and glucated peptides #6a/b (Fig. 3B), as confirmed by MALDI-MS (Fig. S8, A/B). Tandem mass spectrometry confirmed the elution order based on isomer-specific fragmentation patterns (Fig. S8, C/D). Assuming that the separation of glycated peptide isomers was mostly related to neutral conditions, more volatile buffers better suitable for ESI–MS were evaluated. Despite its low buffer capacity at pH 7, ammonium acetate was tested due to its common application in native ESI–MS [30]. Surprisingly, glucated, fructated, and unmodified peptides #2 coeluted at room temperature (*w*_h_ = 0.83 min) and at 60 °C (*w*_h_ = 0.72 min) in a broad asymmetrical peak (Fig. S9). Apparently, phosphate ions favored the separation of glucated and fructated peptides at elevated temperatures. This might be related to stronger interactions of hydroxyl groups in Heyns peptides, as phosphate binds to vicinal hydroxyl groups in sugars [31, 32]. Glycopyranosides can form extended hydrogen bond complexes with organic phosphate anions in an aprotic environment forming a complex with two to three hydroxyl groups [33]. Such strong hydrogen bonding was confirmed for glucose-phosphate buffer systems, which indicated pH-dependent interactions increasing with higher HPO_4_^2−^ concentrations [34]. The 1,2-*trans* diol is a stable bidentate H-bonding motif [35], with especially hydroxyl groups in positions 3 and 4 showing a high interaction potential [33], which should also be the case for the 2-amino-2-deoxygluco/mannopyranosyl form in Heyns peptides. Further polarization by intramolecular NH···OH interactions with the nearby amino group in C-2 position could additionally enhance the binding [36] (Fig. 4).

Next, potassium phosphate was tested due to its better solubility in organic solvents compared to sodium phosphate [37]. At room temperature, all three peptides of peptide family #2 coeluted, while at a column temperature of 60 °C the Heyns peptide (*t*_r_ = 31.4 min) eluted before the coeluting Amadori/unmodified peptides (*t*_r_ = 32.9 min; Fig. S10). The resolution was lower than for the eluent system containing sodium phosphate buffer. Even a new column operated with the same acetonitrile gradient slope (eluent B contained only 40% acetonitrile to improve the solubility of potassium phosphate) improved the separation (*R*_s_ = 1.3) and peak shapes (*w*_h_ = 0.59 min/0.7 min) only slightly. The retention times increased by ~ 2.5 min with all peptides eluting between 26.2 min (peptide #3c) and 46.0 min (peptide #6a) (Fig. S11, Fig. 1D–F; Tab. S1). The elution order of the peptides was confirmed by MALDI-MS. Peaks were broader than in the TFA-containing eluent system with *w*_h_ typically ranging from 0.43 to 0.73 min. Besides for peptide #5, tailing factors of 1.22 to 1.72 indicated reasonable peak shapes with a tendency for tailing. Each group of peptides was well separated with unmodified peptides almost being baseline separated. Compared to eluent system A (0.1% TFA), the elution order of the unmodified peptides changed to #3, #2, #7, #5, #4, and #6. The retention behavior of peptide families #3, #7, and #4 changed the most, which corresponded to the largest shifts in the global peptide charge (Tab. S2). Except for peptide #4a, the elution order of unmodified peptides corresponded to log*D* values calculated for pH 7.4. Similar to eluent system A, unmodified and glucated peptides eluted at similar retention times with deviations typically below 0.2 min, except for peptides #6 and #4, where the unmodified peptides eluted 1.5 min and 0.8 min later, respectively, than the corresponding Amadori peptides. Interestingly, Heyns peptides displayed lower signal intensities in the mass spectra than isomeric Amadori peptides, although similar peak heights and areas were observed by UV detection, most likely due to the phosphate interactions suppressing ionization in positive ion mode, which supports the discussion above. They were partially separated from the Amadori peptides with retention time differences of at least 0.7 min (#7, *R*_s_ = 0.7) and up to 1.6 min (#4, *R*_s_ = 1.5), except for peptide #3 with a shift of only 0.2 min. Thus, medium-sized glycated peptides could be separated independent of the sequence, basicity, and hydrophobicity. Despite its potential to at least partially separate glycation isomers, the phosphate-buffered eluent system suffers from MS compatibility. Furthermore, the column backpressure increased and the resolution decreased over time.

### RP-HPLC–ESI–MS

TFA and phosphate suppress ionization in ESI–MS and can lead to depositions in the ion source [38]. Thus, we tested acetonitrile gradients in the presence of formic acid (0.1% v/v). A fast gradient using a slope of 1.8% CH_3_CN per minute eluted the glycated peptides between 12 and 21 min with ESI–MS confirming the same elution order as in the presence of TFA (Fig. S13). Except the partially separated glycated peptides #2 and #3, all other peptides of a given modification were baseline separated, which was further improved for a slope of 0.6% per minute (Fig. 1G and H, Tab. S1). Peaks were narrow (*w*_h_ = 0.16 to 0.42 min), but showed a strong tailing (*T*_10%_ = 1.59–2.51). The retention times of glycated peptide isomers were very similar with those of fructated peptides eluting ~ 0.1 to 0.3 min earlier and coeluted as broad peaks (*w*_h_ = 0.36–0.72 min) when all 20 peptides were analyzed (Fig. S15).

**Fig. 5:**
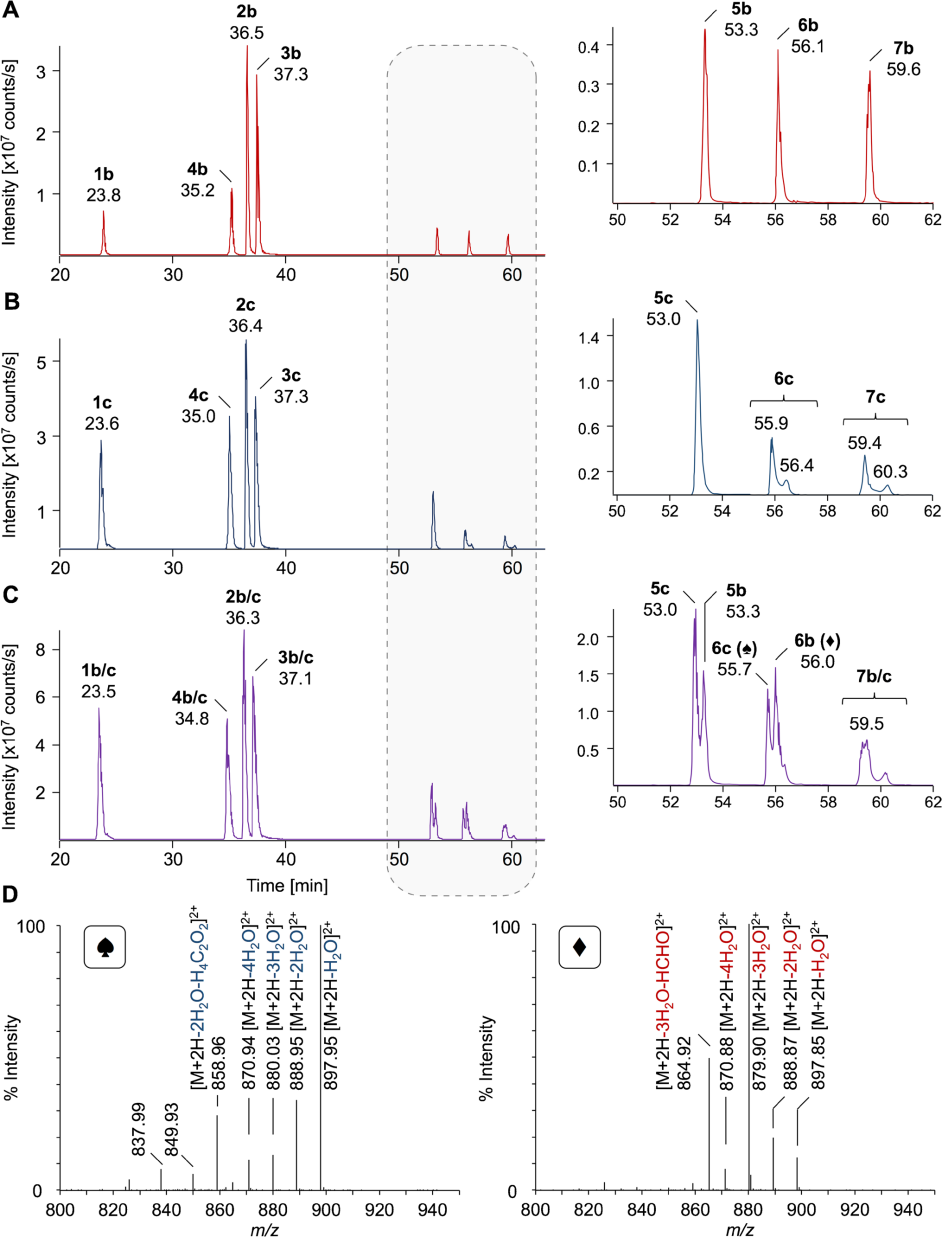
Extracted ion chromatograms (XICs) of triply (peptides #6, #2, #5, #4, and #7) and quadruply (peptides #3 and #1) protonated precursor ions of glucated peptides (b; panel A), fructated peptides (c; panel B), and a mixture of glycated peptides (panel C; 500 fmol each). Peptides were separated on a nanoRP-UPLC-ESI-Orbitrap-MS using an Acquity UPLC BEH C_18_ column (30 °C) and a linear 87-min gradient from 3 to 40% aqueous acetonitrile containing 0.1% formic acid. The inserts display the XICs from 50 to 62 min. Tandem mass spectra (panel D) recorded for the doubly protonated precursor ions at m/z 906.98 eluting at 55.7 min (6c) and 56.0 min (6b) when separating the glycated peptide mixture (panel C) confirming the structures of Amadori and Heyns peptides, respectively. Peptide sequences and modification sites are provided in Table 1

An Acquity UPLC BEH C_18_ column consisting of ethylene bridged hybrid particles (1.7 µm) with (trifunctional) bonded octadecyl chains [39] operated with a shallow linear gradient (30 °C) was tested online to an ESI-Orbitrap-MS, conditions we typically use for bottom-up proteomics [40]. XICs generated for the most intense quasimolecular ions of all Amadori peptides indicated a similar separation as the Jupiter C_18_ column (Fig. 5A and Fig. 1G). Only peptide #4 eluted earlier before peptide #2. The corresponding Heyns peptides eluted up to 0.3 min earlier (Fig. 5B). Glycated peptide isomers typically coeluted as sharp symmetrical peaks (Amadori: *w*_h_ = 0.13 to 0.22 min; Heyns: *w*_h_ = 0.19 to 0.32 min). However, late-eluting fructated peptides #6c and #7c were partially separated with retention time differences of 0.5 min and 0.9 min (*R*_s_ = 2.4), respectively. The characteristic fragmentation pattern confirmed fructated peptides in both peaks and excluded a glucated peptide (data not shown) [14]. These peaks may represent tautomeric forms, i.e., α- or β-pyranosyl forms, or more likely epimers, i.e., glucosyl-/mannosyllysine considering a previous report [23]. The increased resolution of late-eluting glycated peptides was also observed for isomeric Amadori and Heyns peptides (Fig. 5C). For example, glucated peptide #6b (*t*_r_ = 56.0 min) eluted between the fructose-derived glucosyl- (*t*_r_ = 55.7 min) and mannosyl-lysine-containing peptides #6c (*t*_r_ = 56.3 min), corresponding to a chromatographic resolution of 1.1 for the first two species. Fructated and glucated peptides #5 were decently separated (*R*_s_ = 1.0). Similar patterns were detected for glycated peptides #7, but glucosyl- and fructosyl-lysine-derived peptides coeluted in a broad peak (*t*_r_ = 59.5 min, *w*_h_ = 0.4), while the mannosyl species was separated (*t*_r_ = 60.2 min, *R*_s_ = 1.3).

### Hydrophilic interaction chromatography

**Fig. 6:**
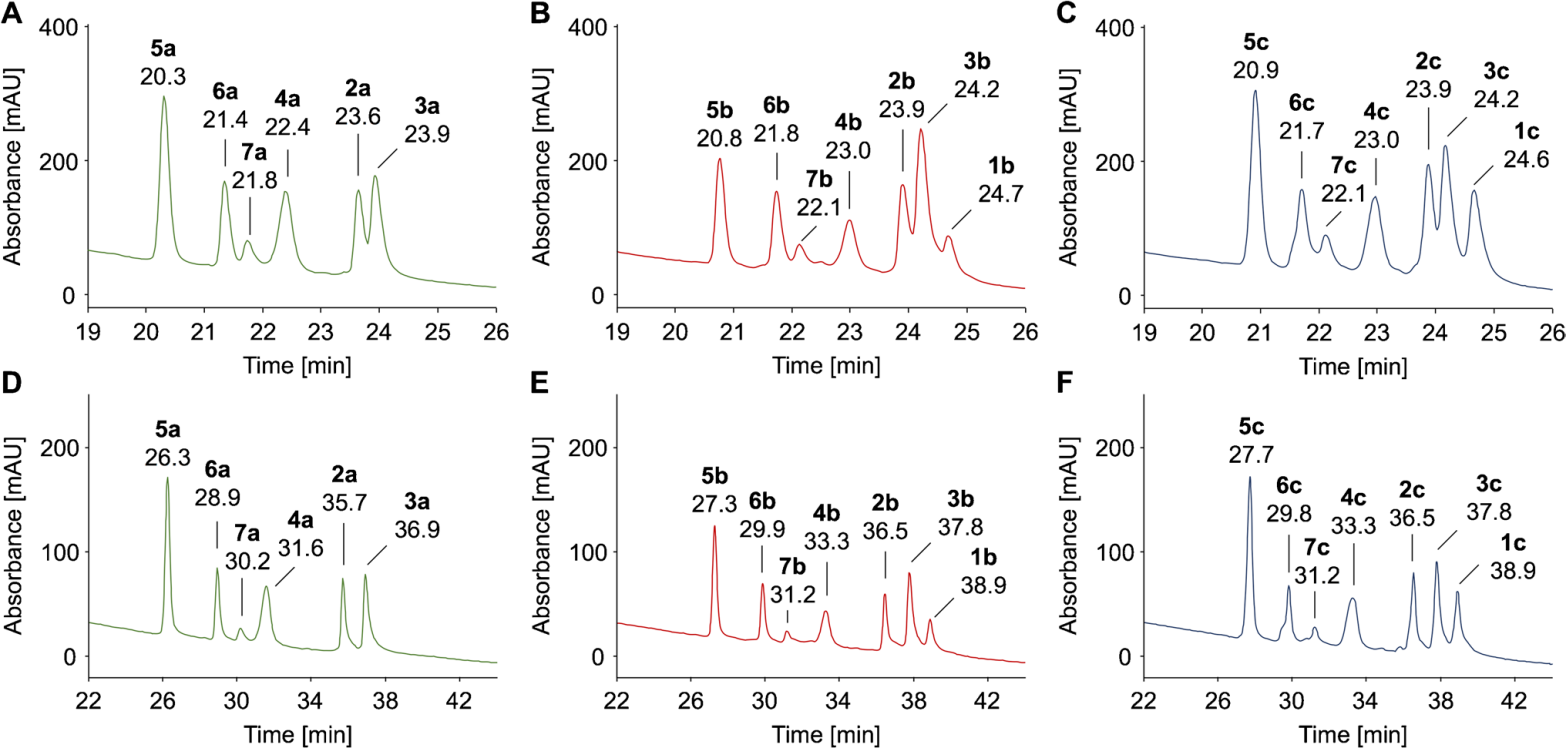
HILIC chromatograms of unmodified (panel A, D), glucated (panel B, E), and fructated peptides (panel C, F) (500 pmol each) displayed from 19 to 26 min or from 22 to 43 min. Peptides were separated on a Luna-HILIC column at room temperature using eluent system E and gradients with a slope of 1.8% (panels A–C) or 0.6% water per minute (panels D–F). Absorbance was recorded at 214 nm. Full chromatograms are provided in the Supplement (Fig. S16/S18). Peptide sequences and modification sites are provided in Table 1

HILIC can be favorably coupled to ESI–MS when using volatile eluents, such as aqueous acetonitrile. The present study used a silica-bonded cross-linked diol stationary phase, which provides a high stability due to reduced irreversible adsorption and the absence of Schiff base formation by reducing sugars [41]. Retention relies on hydrogen donor and acceptor capabilities, electrostatic and hydrophobic interactions, and hydrophilic partitioning [16]. Negative log*P* and log*D* values calculated for pH 3.2 indicated that all peptides are sufficiently polar for the HILIC separation mode (Table 1) [42]. However, all peptides eluted in a narrow range from 20 to 25 min with glucated, fructated, and unmodified peptides separated in sharp peaks (*w*_h_ = 0.17 to 0.28 min) using a water gradient (slope of 1.8% per min) at room temperature. The peptides were only partially baseline separated when injected as mixtures (Fig. 6A–C; Tab. S1) with late-eluting peptides #2, #3, and #1 only partially resolved (*R*_s_ = 0.8 to 1.2). The elution order of the peptide families was always #5, #6, #7, #4, #2, #3, and #1 independent of the peptide modification (Fig. S17), and thus basically reversed to RP-HPLC. However, some peptides switched the elution order, most likely due to the intermediate pH used in HILIC. Partition coefficients at pH 3.2 strongly correlated with experimental retention times (Spearman rank correlation coefficients *r*_S_ =  − 0.95), well predicting retention. Glycated peptides eluted always later than the corresponding unmodified peptides, which reflects the retention of the sugar moiety. Early-eluting unmodified peptides were separated from the corresponding glycated peptide by at least 0.3 min, resulting in nearly baseline separations for peptide families #5 (Δ*t*_r_ = 0.5 min, *R*_s_ = 1.3, Fig. S17A) and #4 (Δ*t*_r_ = 0.6 min, *R*_s_ = 1.3, Fig. S17D). Glucated and the corresponding fructated peptides coeluted. A shallower gradient (0.6% water/min, RT) allowed a baseline separation of all seven glycated and all six unmodified peptides (*R*_s_ ≥ 1.8), while the peak shape increased only slightly (except for #4, *w*_h_ = 0.25 to 0.37 min) (Fig. 6D–F; Tab. S1). Furthermore, the retention times of unmodified and glycated peptides differed by 1 to 1.7 min. However, fructated peptides still coeluted with the corresponding glucated peptides, except for peptide #5. Thus, HILIC is favorable to separate glycated and unmodified peptides, which might be helpful in peptide synthesis, but is generally unable to separate glucated and fructated peptides and is thus not useful for related peptidomics studies. It should be noted that some peptides were less soluble in HILIC eluents due to the high organic content compared to RP-HPLC eluents, especially for peptide #7, which may limit the sensitivity.

## Conclusion

Despite the high relevance of significantly elevated glycated protein levels in diabetes and obesity as an important indicator for the development of secondary diseases, analytical techniques for quantitation of specific glucation and fructation sites in proteins are still missing. Considering a typical bottom-up LC–MS proteomics approach, we have tested eluent systems in RP-HPLC and HILIC suitable for online ESI–MS for the separation of seven glucated tryptic peptides from the corresponding isomeric fructated peptides. Commonly applied acidic aqueous acetonitrile eluents were able to separate unmodified, glucated, and fructated peptides on a C_18_ column, but glucated, fructated, and typically even the unmodified peptides of a given sequence coeluted. Glycated peptide isomers of hydrophobic sequences were partially separated using shallow gradients at a column temperature of 30 °C in the presence of formic acid. HILIC separated unmodified and glycated peptides, but glucated and fructated peptides coeluted. The best separation was achieved in RP-HPLC using neutral, acetonitrile-containing eluent systems; phosphate-buffered eluents especially were able to separate most glucated and fructated peptide isomers. Fructated peptides typically eluted before the corresponding glucated peptide equivalent to a difference in the eluent composition of 1% acetonitrile. As the eluent system is only partially compatible with LC–ESI–MS, it might be more favorable to use it as the first dimension in two-dimensional LC–ESI–MS.

## Supplementary Information

Below is the link to the electronic supplementary material.

Supplementary file1 (PDF 3318 KB)
