# Supplementary material for: Chromatographic separation of glycated peptide isomers derived from glucose and fructose
Source: Anal Bioanal Chem. 2022 Aug 3;414(23):6801–12. doi: 10.1007/s00216-022-04243-9 (PMC9436859; doi:10.1007/s00216-022-04243-9)
Supplement: Supplementary file 1 — Supplementary file1 (PDF 3318 KB) [file 216_2022_4243_MOESM1_ESM.pdf]

Supporting Information

Chromatographic separation of glycated peptide isomers derived from glucose and fructose

Sebastian Schmutzler[1,2] and Ralf Hoffmann[1,2]*

[1] Institut für Bioanalytische Chemie, Fakultät für Chemie und Mineralogie, Universität Leipzig, Leipzig, Germany

[2] Biotechnologisch-Biomedizinisches Zentrum, Universität Leipzig, Leipzig, Germany

* Corresponding authors

Prof. Dr. Ralf Hoffmann

Institut für Bioanalytische Chemie

Biotechnologisch-Biomedizinisches Zentrum

Deutscher Platz 5, 04103 Leipzig, Germany

E-mail: bioanaly@rz.uni-leipzig.de

Table S1. Retention times of synthetic peptides in RP-HPLC and HILIC using different eluent systems.

#	Protein/ Location		Sequence	RP-LC-t_R (TFA, 60°C)	RP-LC-t_R (K-PBS, 60°C)	RP-LC-t_R (FA, 60°C)	HILIC-t_R (NH_4HCOO, RT)	
				1% A2 [min]	1.5% B3 [min]	1%C2 [min]	4.5% E2 [min]	1.5% E2 [min]
1	HP A$_{77}$-K$_{93}$ **K**$_{81}$	B	AVGD**K**$_{Ama}$LPEC*EAVC*GKPK	27.8	29.9	23.6	24.7	38.9
		C	AVGD**K**$_{Hey}$LPEC*EAVC*GKPK	27.6	28.9	23.4	24.7	38.9
2	HSA A$_{258}$-K$_{274}$ **K**$_{262}$	A	ADLAKYIC*ENQDSISSK	35.0	33.9		23.6	35.7
		B	ADLA**K**$_{Ama}$YIC*ENQDSISSK	34.7	33.7	31.5	23.9	36.5
		C	ADLA**K**$_{Hey}$YIC*ENQDSISSK	34.6	32.6	31.4	23.9	36.5
3	HSA T$_{52}$-K$_{73}$ **K**$_{64}$	A	TC*VADESAENC*DKSLHTLFGDK	37.2	26.4		23.9	36.9
		B	TC*VADESAENC*D**K**$_{Ama}$SLHTLFGDK	36.6	26.4	32.4	24.2	37.8
		C	TC*VADESAENC*D**K**$_{Hey}$SLHTLFGDK	36.7	26.2	32.3	24.2	37.8
4	HSA K$_{414}$-K$_{428}$ **K**$_{414}$	A	KVPQVSTPTLVEVSR	38.7	44.4		22.4	31.6
		B	**K**$_{Ama}$VPQVSTPTLVEVSR	38.2	43.6	33.8	23.0	33.3
		C	**K**$_{Hey}$VPQVSTPTLVEVSR	38.3	42.0	33.7	23.0	33.3
5	HSA V$_{373}$-K$_{389}$ **K**$_{378}$	A	VFDEFKPLVEEPQNLIK	49.2	42.4		20.3	26.3
		B	VFDEF**K**$_{Ama}$PLVEEPQNLIK	49.1	42.4	45.9	20.8	27.3
		C	VFDEF**K**$_{Hey}$PLVEEPQNLIK	48.9	41.0	45.6	20.9	27.7
6	HSA A$_{226}$-K$_{240}$ **K**$_{233}$	A	AEFAEVSKLVTDLTK	52.5	46.0		21.4	28.9
		B	AEFAEVS**K**$_{Ama}$LVTDLTK	52.1	44.5	47.0	21.8	29.9
		C	AEFAEVS**K**$_{Hey}$LVTDLTK	51.8	43.3	46.8	21.7	29.8
7	HSA E$_{542}$-K$_{557}$ **K**$_{545}$	A	EQLKAVMDDFAAFVEK	53.4	37.3		21.8	30.2
		B	EQL**K**$_{Ama}$AVMDDFAAFVEK	52.6	37.4	48.6	22.1	31.2
		C	EQL**K**$_{Hey}$AVMDDFAAFVEK	52.3	36.7	48.4	22.1	31.2

Table S2. Net charges of peptide standards at different pH (2.0, 3.2, 7.2) calculated by MarvinSketch (Product version 18.30.0).

#	Protein/ Location	Sequence	Charge at pH 2.0	Charge at pH 3.2	Charge at pH 7.2
1	HP<br>A_{77}-K_{93}<br>$\mathbf{K}_{81}$	AVGDKLPEC*EAVC*GKPK	+3.9	+3.0	-0.1
		AVGD$\mathbf{K}_{\mathrm{Hex}}$LPEC*EAVC*GKPK	+3.9	+3.0	-0.2
2	HSA<br>A_{258}-K_{274}<br>$\mathbf{K}_{262}$	ADLAKYIC*ENQDSISSK	+2.9	+1.9	-1.1
		ADLA$\mathbf{K}_{\mathrm{Hex}}$YIC*ENQDSISSK	+2.9	+1.9	-1.2
3	HSA<br>T_{52}-K_{73}<br>$\mathbf{K}_{64}$	TC*VADESAENC*DKSLHTLFGDK	+3.8	+2.3	-3.0
		TC*VADESAENC*D$\mathbf{K}_{\mathrm{Hex}}$SLHTLFGDK	+3.8	+2.1	-3.1
4	HSA<br>K_{414}-K_{428}<br>$\mathbf{K}_{414}$	KVPQVSTPTLVEVSR	+3.0	+2.7	+0.9
		$\mathbf{K}_{\mathrm{Hex}}$VPQVSTPTLVEVSR	+3.0	+2.7	+0.8
5	HSA<br>V_{373}-K_{389}<br>$\mathbf{K}_{378}$	VFDEFKPLVEEPQNLIK	+2.9	+1.8	-2.1
		VFDEF$\mathbf{K}_{\mathrm{Hex}}$PLVEEPQNLIK	+2.9	+1.8	-2.2
6	HSA<br>A_{226}-K_{240}<br>$\mathbf{K}_{233}$	AEFAEVSKLVTDLTK	+2.9	+2.2	-1.1
		AEFAEVS$\mathbf{K}_{\mathrm{Hex}}$LVTDLTK	+2.9	+2.2	-1.2
7	HSA<br>E_{542}-K_{557}<br>$\mathbf{K}_{545}$	EQLKAVMDDFAAFVEK	+2.9	+1.8	-2.1
		EQL$\mathbf{K}_{\mathrm{Hex}}$AVMDDFAAFVEK	+2.9	+1.8	-2.2

Hex, hexosamine-modified lysine; * carbamidomethylation with iodoacetamide

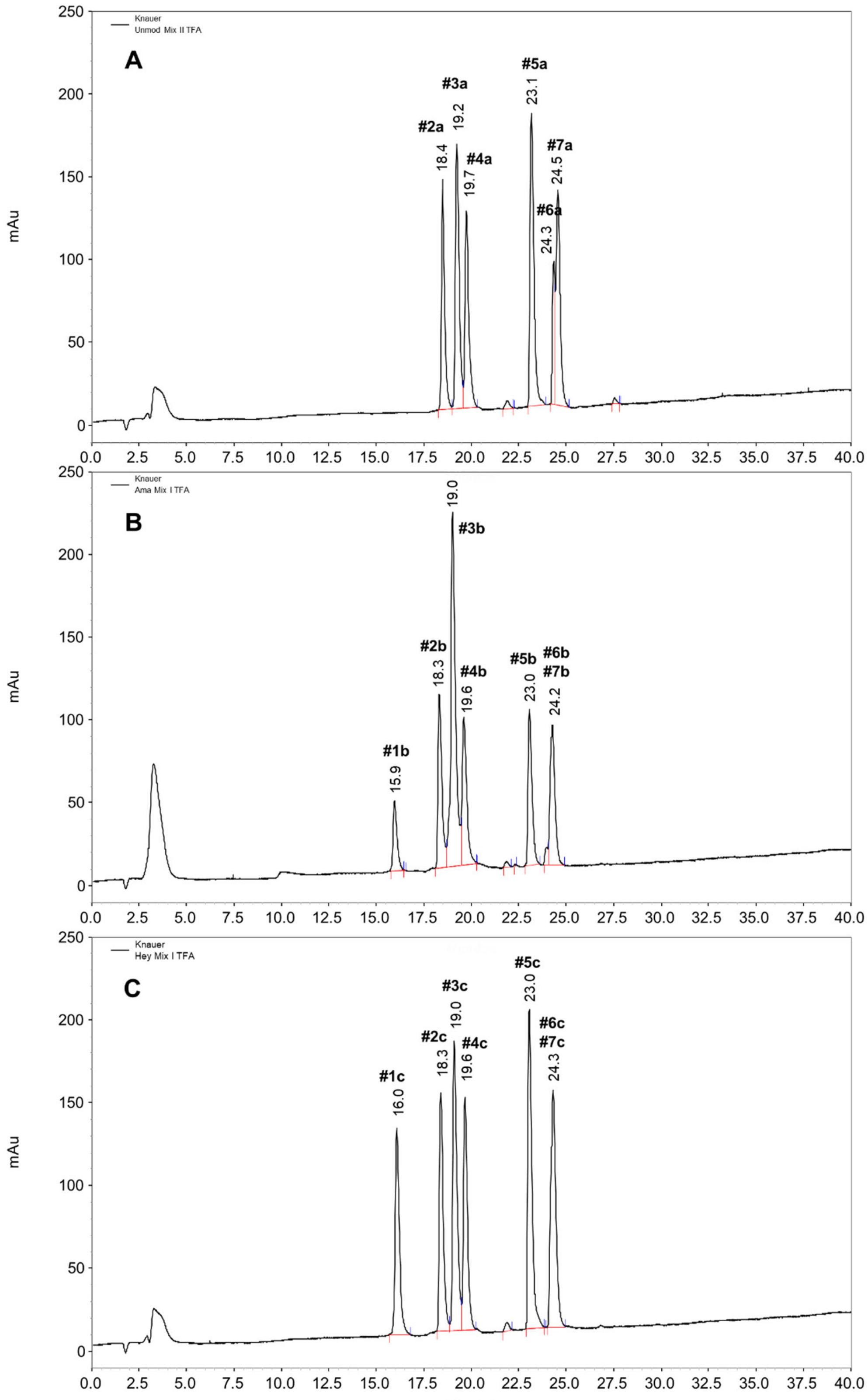

Figure S1: RP chromatograms of unmodified (A, n=6), glucated (B, n=7) and fructated (C, n=7) peptides (each 500 pmol). Compounds were analyzed by RP-HPLC on a Jupiter C_{18}-column using a linear 30-min gradient from 3% to 57% aqueous acetonitrile (1.8% CH_3CN per min) containing 0.1% TFA. Separations were performed at 60 °C and the absorbance was recorded at 214 nm. Peptide sequences and modification sites are provided in Tab. 1.

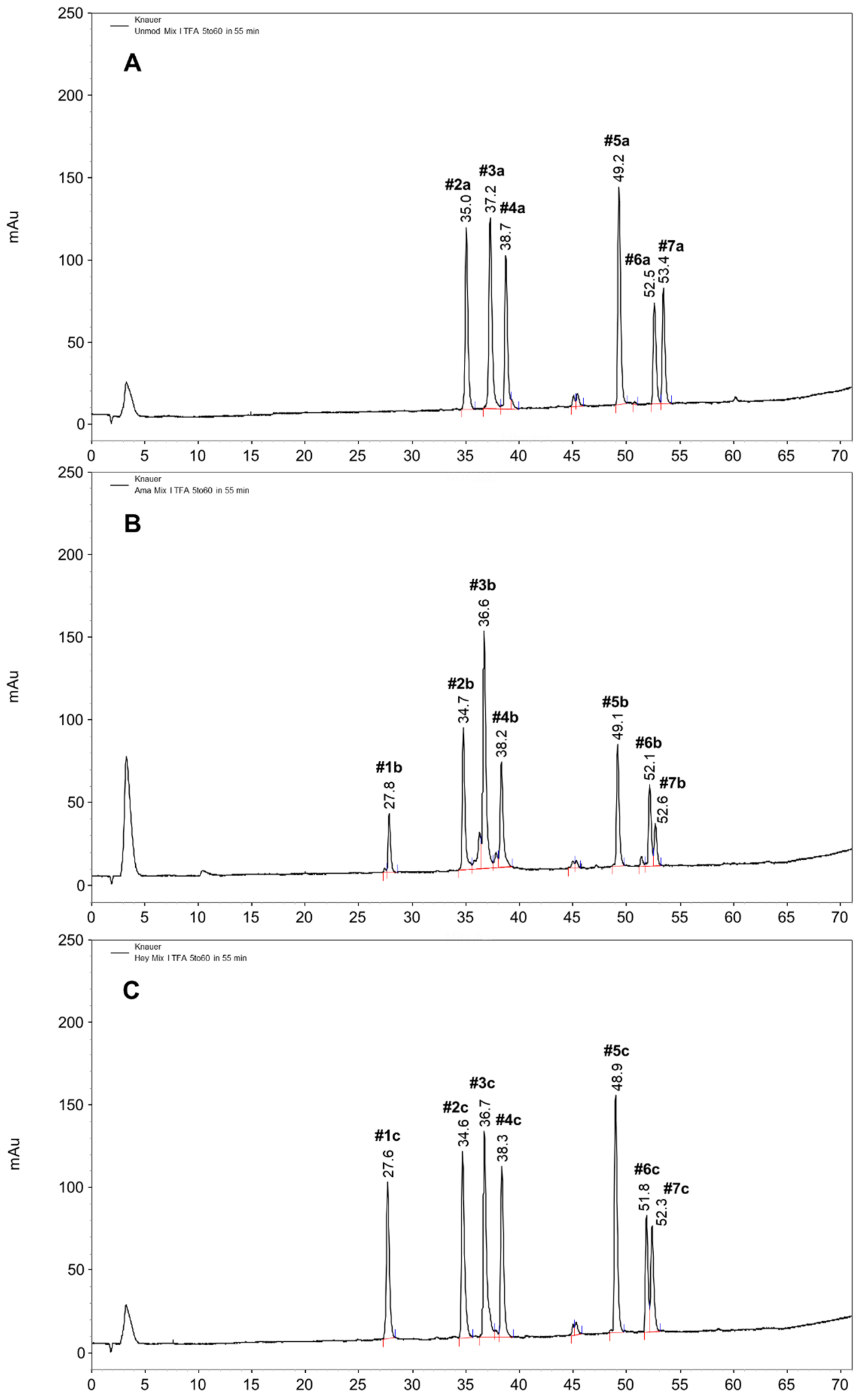

Figure S2: RP chromatograms of unmodified (A, n=6), glucated (B, n=7) and fructated (C, n=7) peptides (each 500 pmol). Compounds were analyzed by RP-HPLC on a Jupiter C_{18}-column using a linear 55-min gradient from 3% to 36% aqueous acetonitrile (0.6% CH_3CN per min) containing 0.1% TFA. Separations were performed at 60 °C and the absorbance was recorded at 214 nm. Peptide sequences and modification sites are provided in Tab. 1.

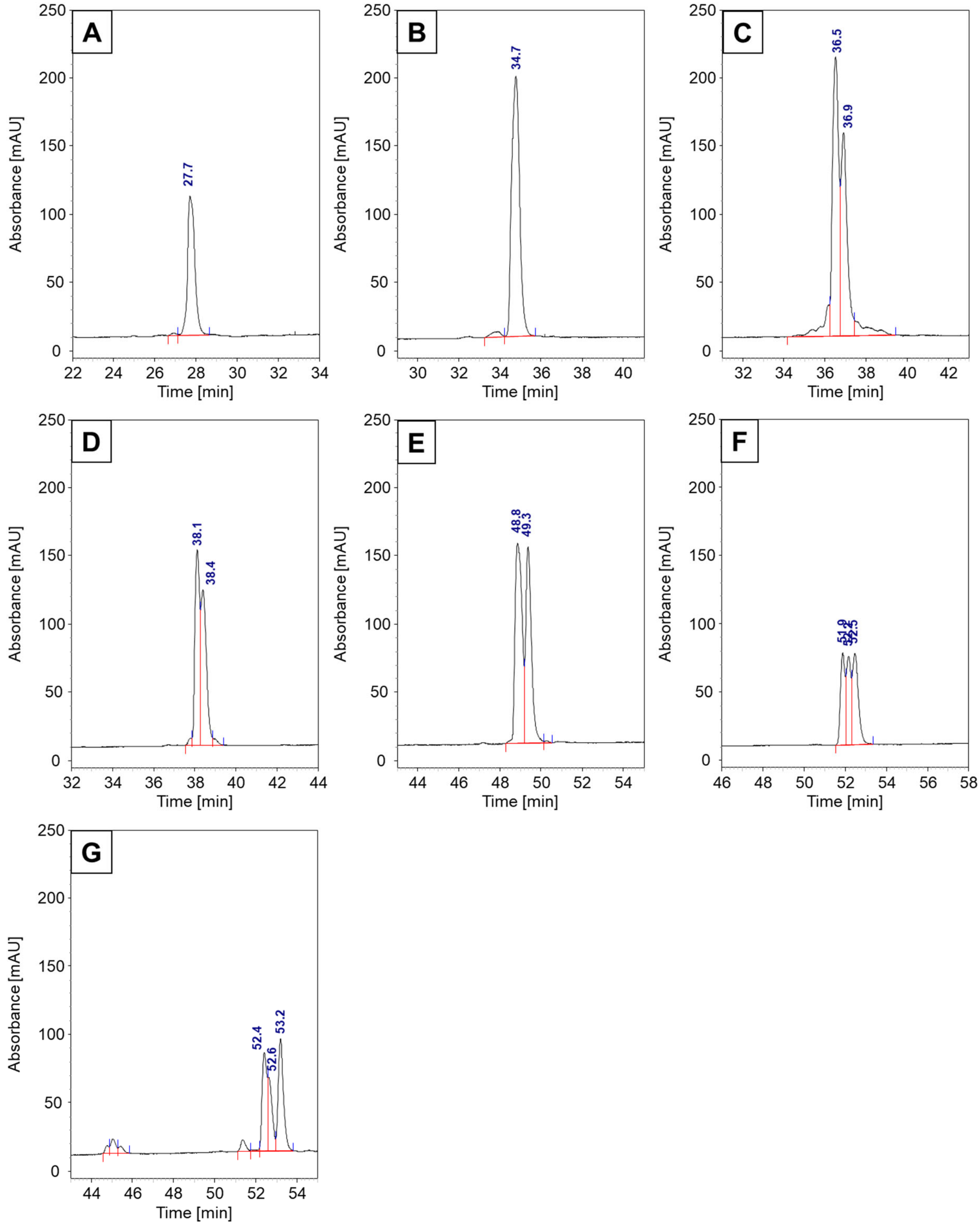

Figure S3: Sections of RP chromatograms of peptide families #1 (A), #2 (B), #3 (C), #4 (D), #5 (E), #6 (F), and #7 (G) consisting of glucated, fructated, and unmodified (except #1) peptides (500 pmol each). Peptide mixtures were analyzed on a Jupiter C_{18}-column (60 °C) using a linear 55-min gradient from 3% to 36% aqueous acetonitrile containing 0.1% TFA. Absorbance was recorded at 214 nm. Full chromatograms are provided in the Supplement (Fig. S4). Peptide sequences and modification sites are provided in Tab. 1.

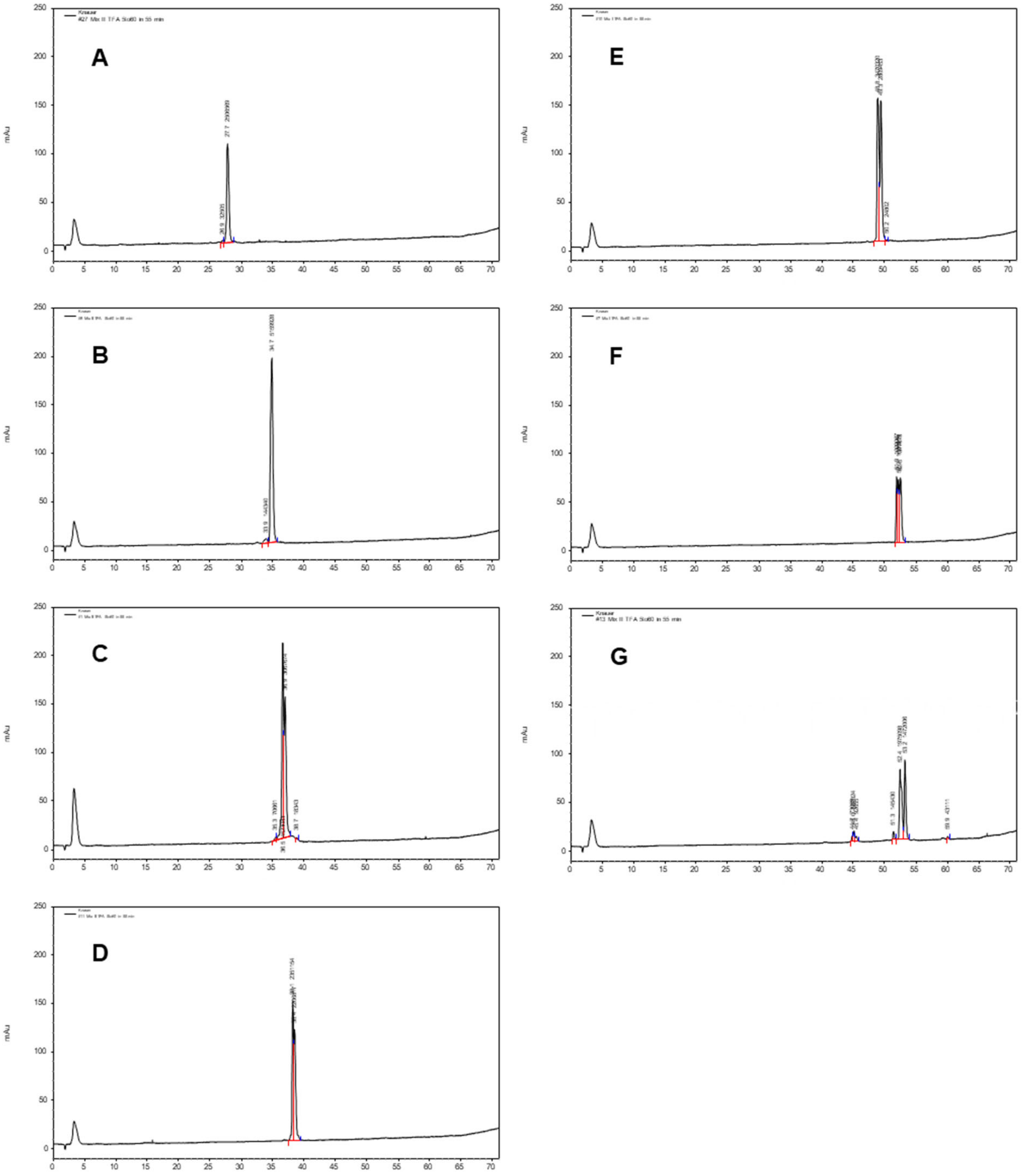

Figure S4: RP chromatograms of peptide families #1 (A), #2 (B), #3 (C), #4 (D), #5 (E), #6 (F), and #7 (G) consisting of glucated, fructated, and unmodified (except #1) peptides (500 pmol each). Peptide mixtures were analyzed on a Jupiter C_{18}-column (60 °C) using a linear 55-min gradient from 3% to 36% aqueous acetonitrile containing 0.1% TFA. Absorbance was recorded at 214 nm. Peptide sequences and modification sites are provided in Tab. 1.

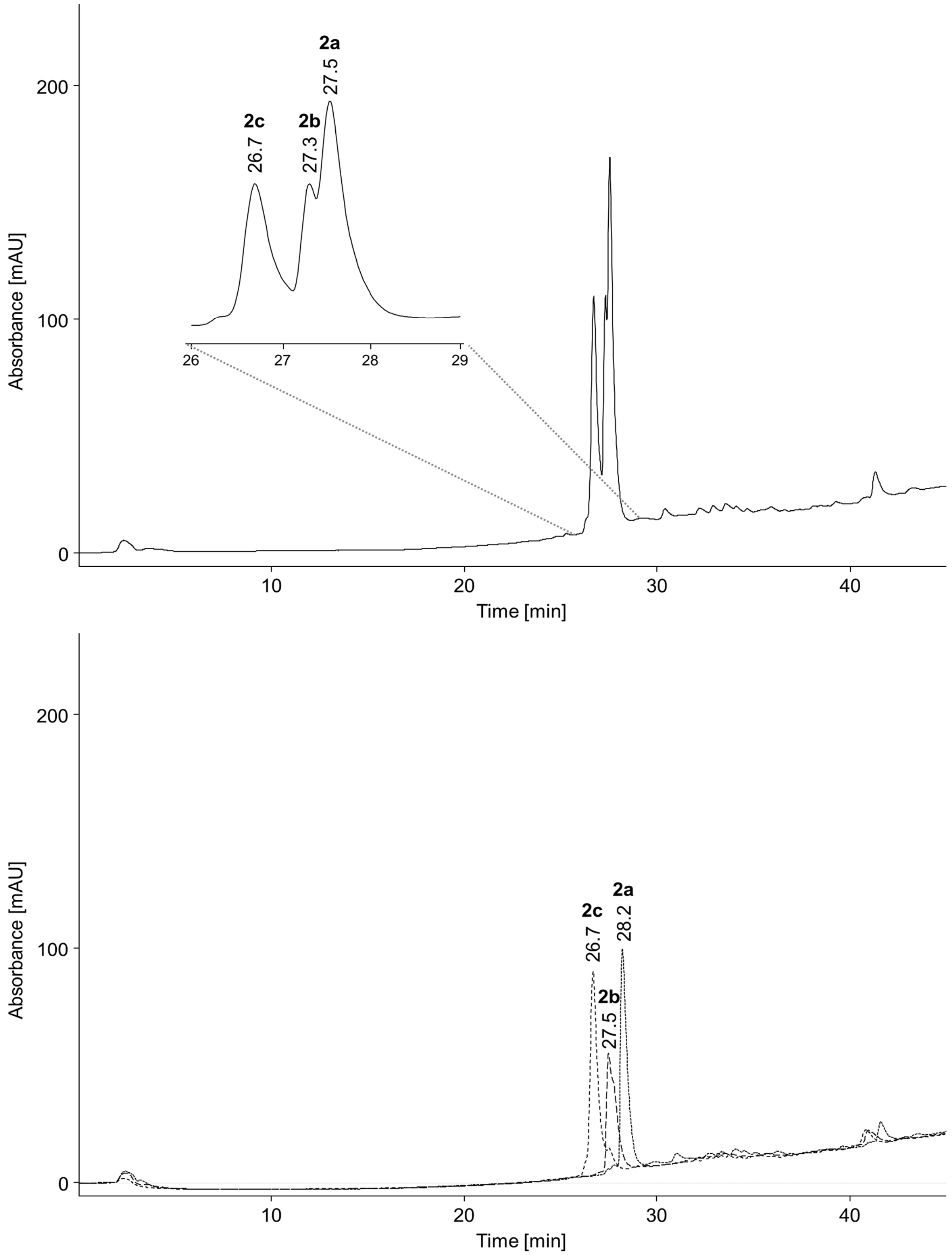

Figure S5: RP chromatograms of unmodified (a), glucated (b) and fructated (c) peptides (500 pmol each) of peptide family #2 (top) and overlay of individual injections (bottom). Compounds were analyzed by RP-HPLC on an Aqua C_{18}-column using a linear 30-min gradient from 3% to 57% aqueous acetonitrile (1.8% CH_3CN per min) containing sodium phosphate buffer (10 mmol/L, pH 7.2). Separations were performed at 60 °C and the absorbance was recorded at 214 nm. Peptide sequences and modification sites are provided in Tab. 1.

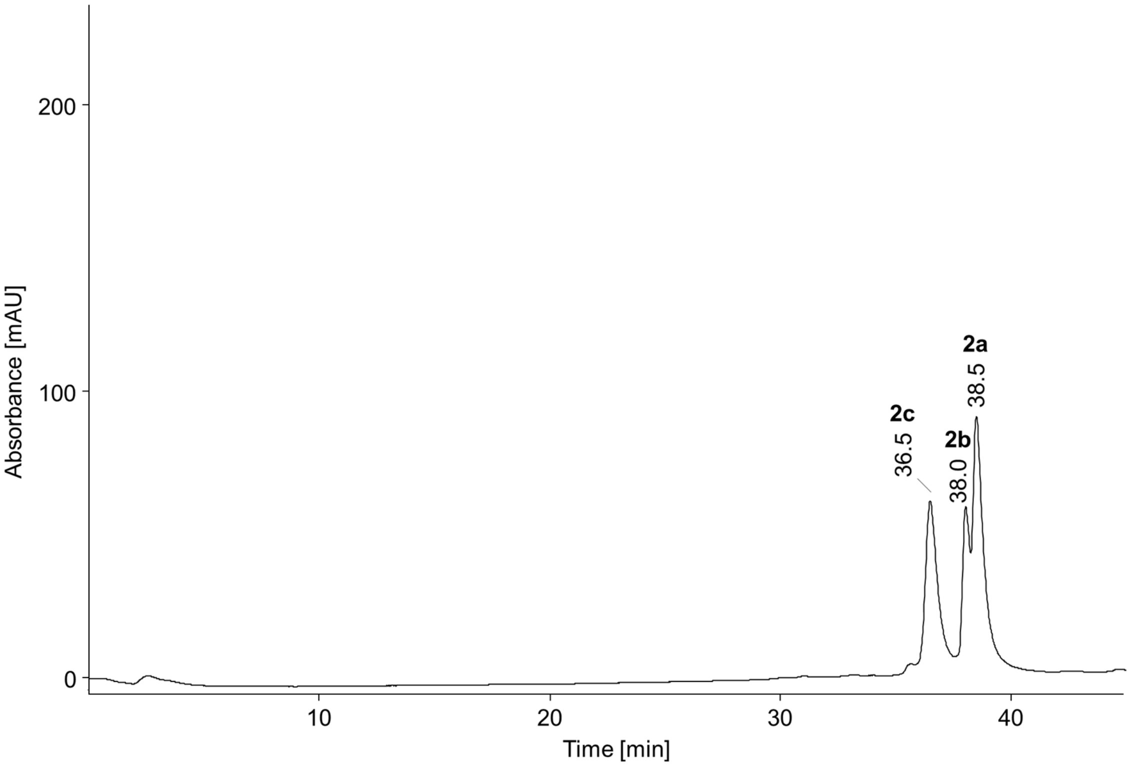

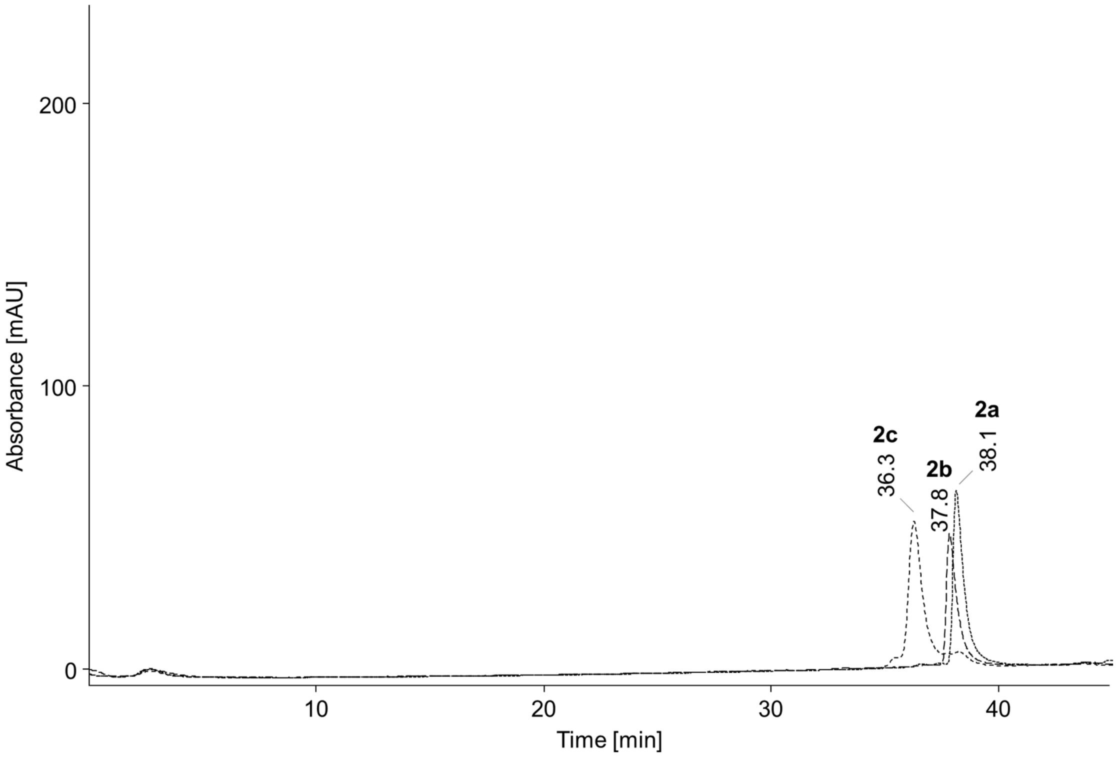

Figure S6: RP chromatograms of unmodified (a), glucated (b) and fructated (c) peptides (500 pmol each) of peptide family #2 (top) and overlay of individual injections (bottom). Compounds were analyzed by RP-HPLC on an Aqua C_{18}-column using a linear 65-min gradient from 3% to 42% aqueous acetonitrile (0.6% CH_3CN per min) containing sodium phosphate buffer (10 mmol/L, pH 7.2). Separations were performed at 60 °C and the absorbance was recorded at 214 nm. Peptide sequences and modification sites are provided in Tab. 1.

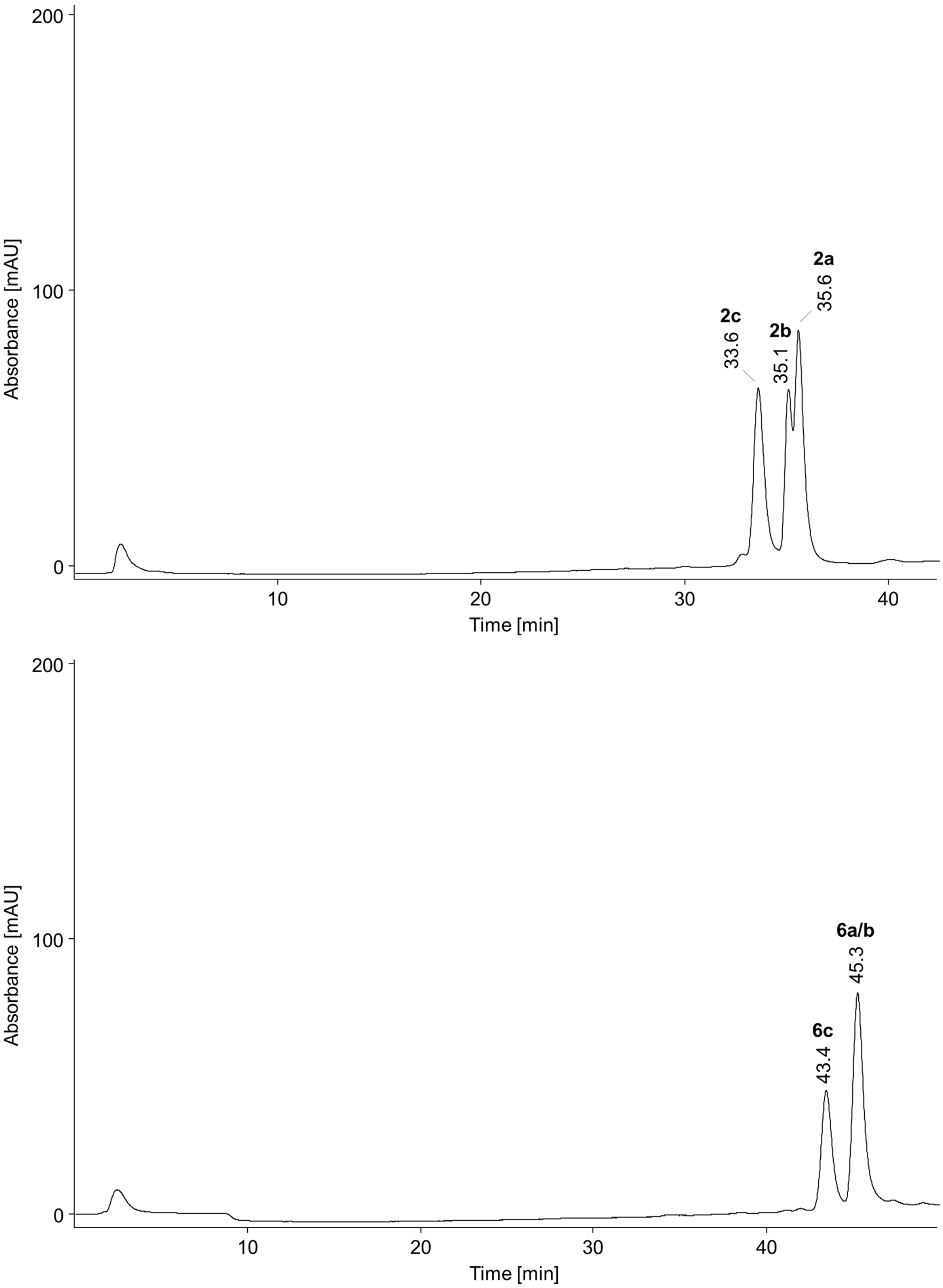

Figure S7: RP chromatograms of unmodified (a), glucated (b) and fructated (c) peptides (500 pmol each) of peptide families #2 (top) and #6 (bottom). Compounds were analyzed by RP-HPLC on a Synergi Fusion RP column using a linear 65-min gradient from 3% to 42% aqueous acetonitrile (0.6% CH_3CN per min) containing sodium phosphate buffer (10 mmol/L, pH 7.2). Separations were performed at 60 °C and the absorbance was recorded at 214 nm. Peptide sequences and modification sites are provided in Tab. 1.

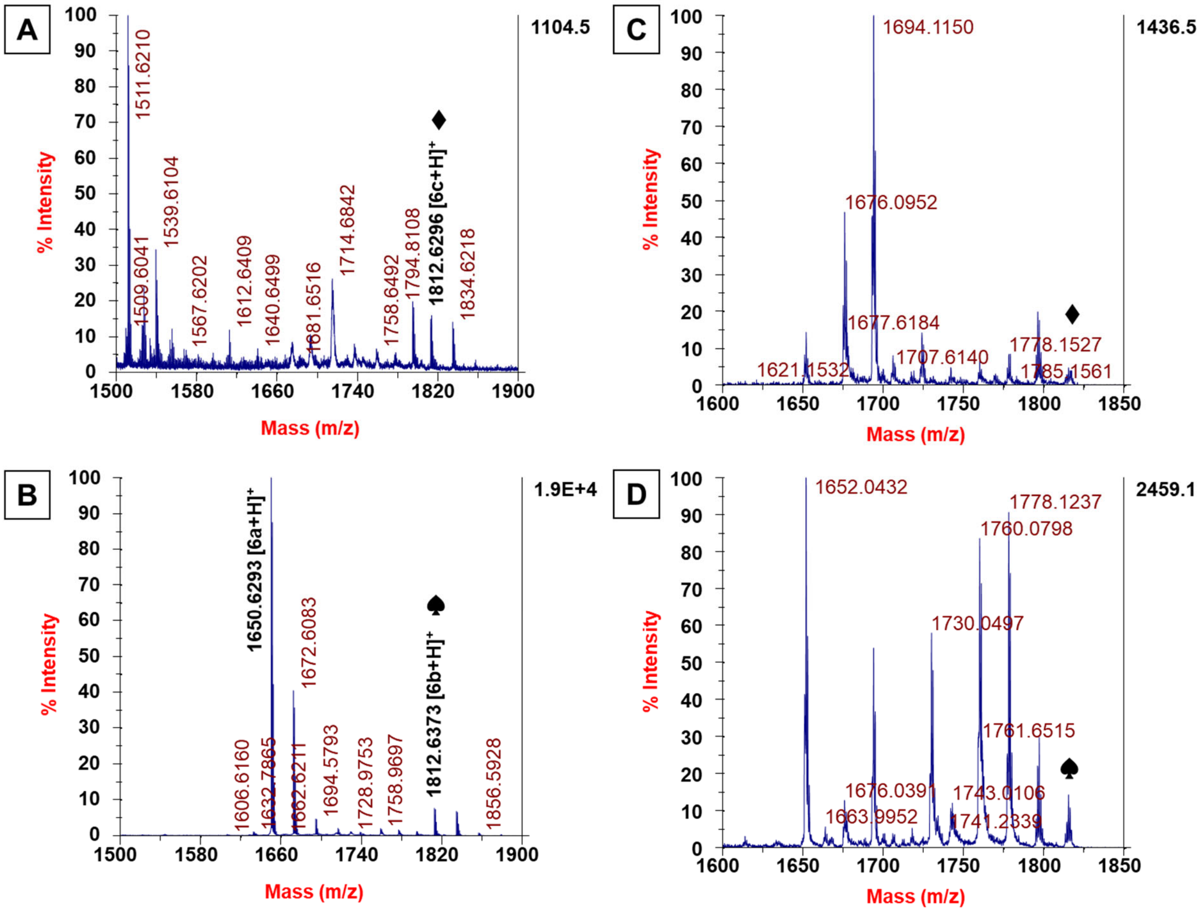

Figure S8: Mass spectra (A, B) of fractions eluting from analytical RP-HPLC of peptide #6 (Fig. 6) and tandem mass spectra of protonated precursor ions (C, D; marked by symbols) recorded on a MALDI-TOF/TOF-MS reveal sugar specific fragmentation patterns.

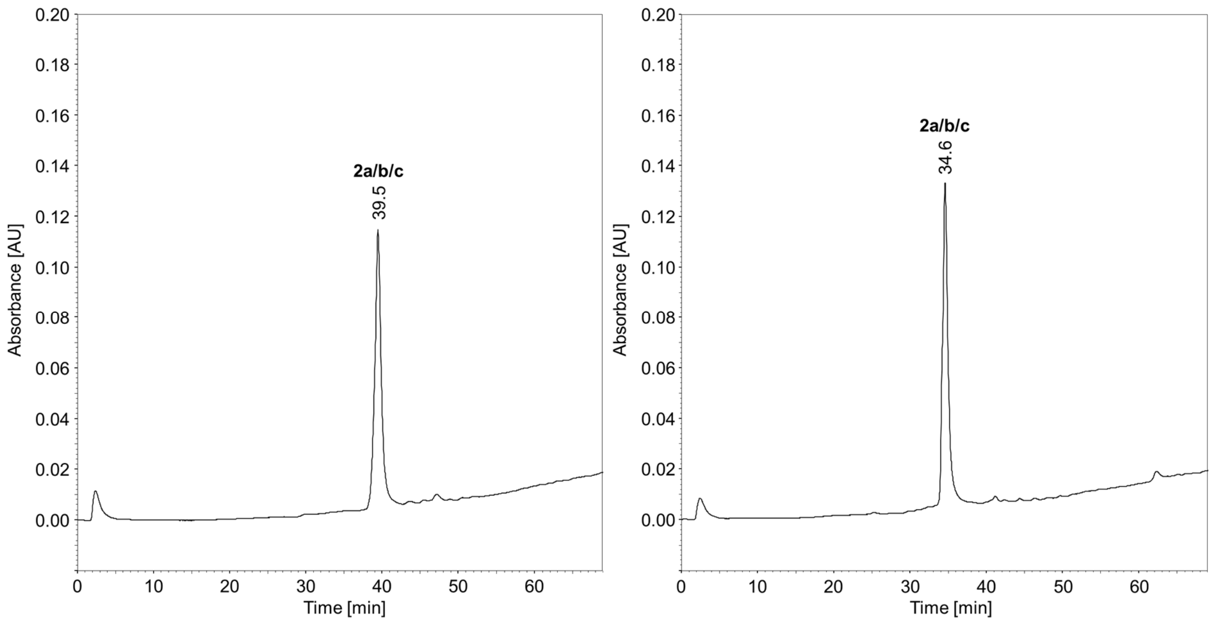

Figure S9: RP chromatograms of unmodified (a), glucated (b) and fructated (c) peptides (each 500 pmol) of peptide family #2. Compounds were analyzed by RP-HPLC on a Synergi Fusion RP-column using a linear 65-min gradient from 3% to 42% aqueous acetonitrile (0.6% CH_3CN per min) containing ammonium acetate (10 mmol/L, pH 7.2). Separations were performed at room temperature (left) or 60 °C (right) and the absorbance was recorded at 214 nm. Peptide sequences and modification sites are provided in Tab. 1.

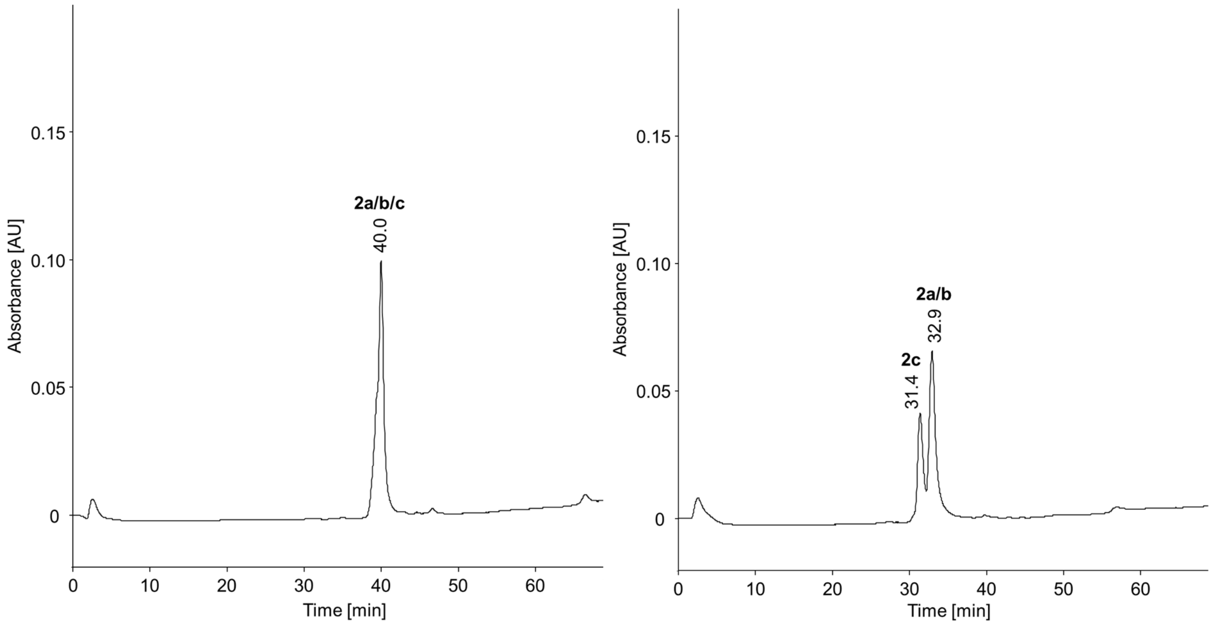

Figure S10: RP chromatograms of unmodified (a), glucated (b) and fructated (c) peptides (500 pmol each) of peptide family #2. Compounds were analyzed by RP-HPLC on a Synergi Fusion RP column using a linear 65-min gradient from 3% to 42% aqueous acetonitrile (0.6% CH_3CN per min) containing potassium phosphate (10 mmol/L, pH 7.2). Separations were performed at room temperature (left) or 60 °C (right) and the absorbance was recorded at 214 nm. Peptide sequences and modification sites are provided in Tab. 1.

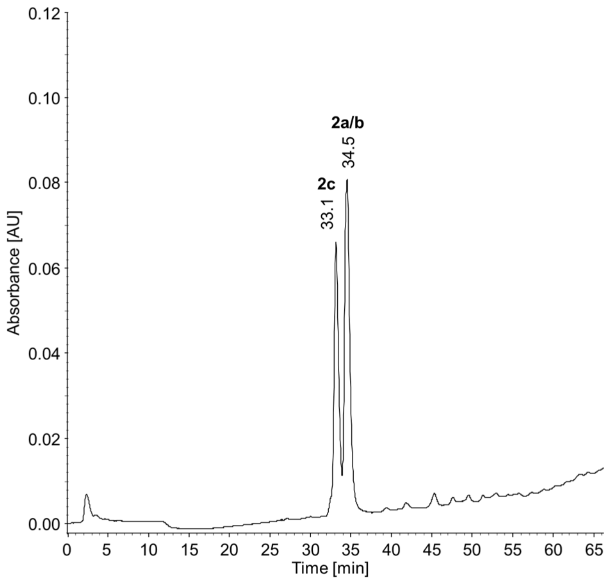

Figure S11: RP chromatogram of unmodified (a), glucated (b) and fructated (c) peptides (500 pmol each) of peptide family #2. Compounds were analyzed by RP-HPLC on a Synergi Fusion RP column using a linear 58-min gradient from 3% to 38% aqueous acetonitrile (0.6% CH_3CN per min) containing potassium phosphate (10 mmol/L, pH 7.2). Separations was performed at 60 °C and the absorbance was recorded at 214 nm. Peptide sequences and modification sites are provided in Tab. 1.

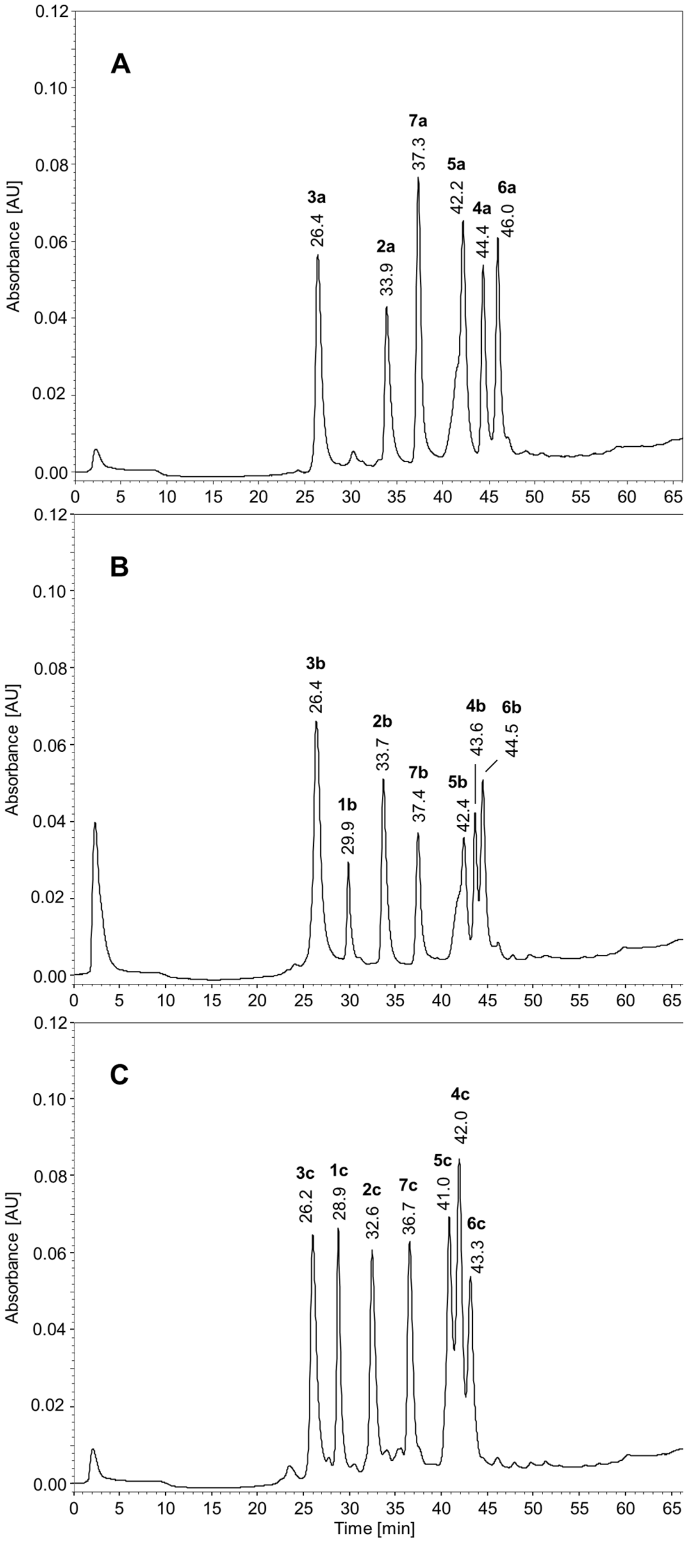

Figure S12: RP chromatograms of unmodified (A, n=6), glucated (B, n=7) and fructated (C, n=7) peptides (500 pmol each). Compounds were analyzed by RP-HPLC on a Synergi Fusion RP column using a linear 58-min gradient from 3% to 38% aqueous acetonitrile (0.6% CH_3CN per min) containing potassium phosphate (10 mmol/L, pH 7.2). Separations was performed at 60 °C and the absorbance was recorded at 214 nm. Peptide sequences and modification sites are provided in Tab. 1.

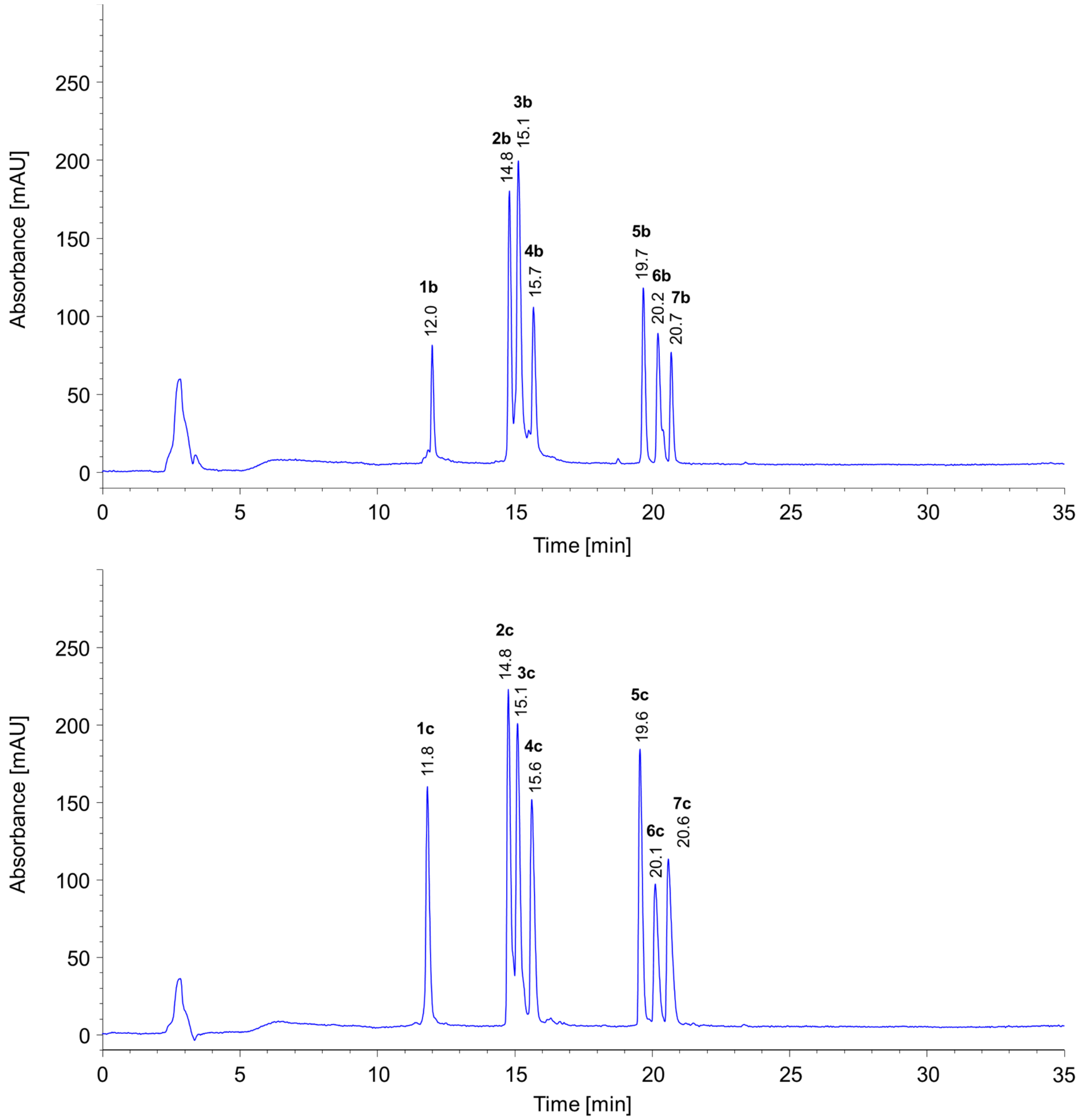

Figure S13: RP chromatograms of glucated (top, each 500 pmol, n=7) and fructated peptides (bottom, 500 pmol each, n=7). Compounds were analyzed by RP-HPLC on a Jupiter C_{18}-column using a linear 30-min gradient from 3% to 57% aqueous acetonitrile (1.8% CH_3CN per min) containing 0.1% formic acid. Separations were performed at 60 °C and the absorbance was recorded at 214 nm. Peptide sequences and modification sites are provided in Tab. 1.

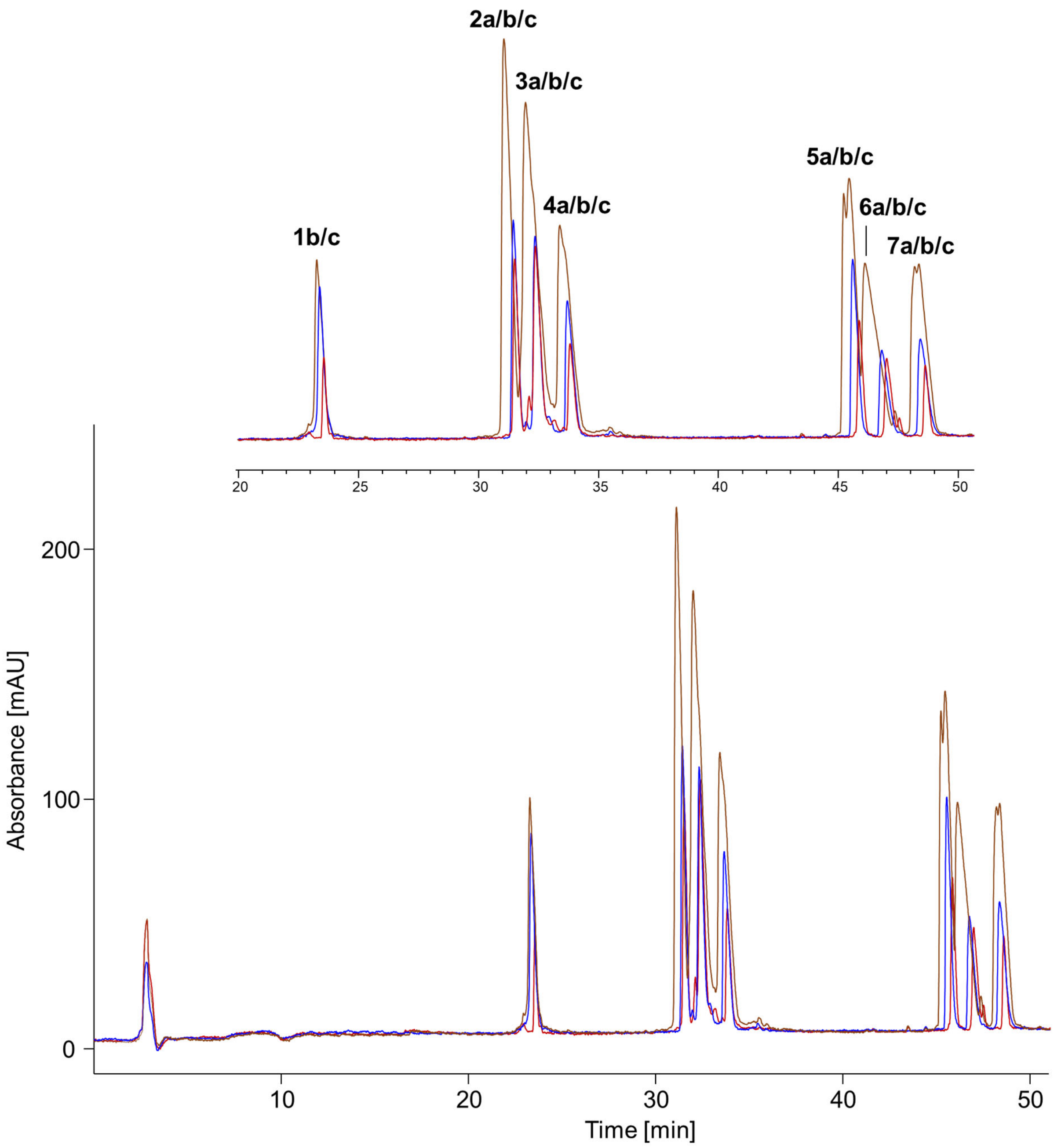

Figure S14: Overlay of RP chromatograms of glucated (red trace, 500 pmol each, n=7). fructated (blue trace, each 500 pmol, n=7) and a mix of glucated, fructated and unmodified peptides (brown trace, each 500 pmol, n=20). Compounds were analyzed by RP-HPLC on a Jupiter C_{18}-column using a linear 55-min gradient from 3% to 36% aqueous acetonitrile (0.6% CH_3CN per min) containing 0.1% formic acid. Separations were performed at 60 °C and the absorbance was recorded at 214 nm. The insert displays the zoomed chromatogram from 20 min to 50 min. Peptide sequences and modification sites are provided in Tab. 1.

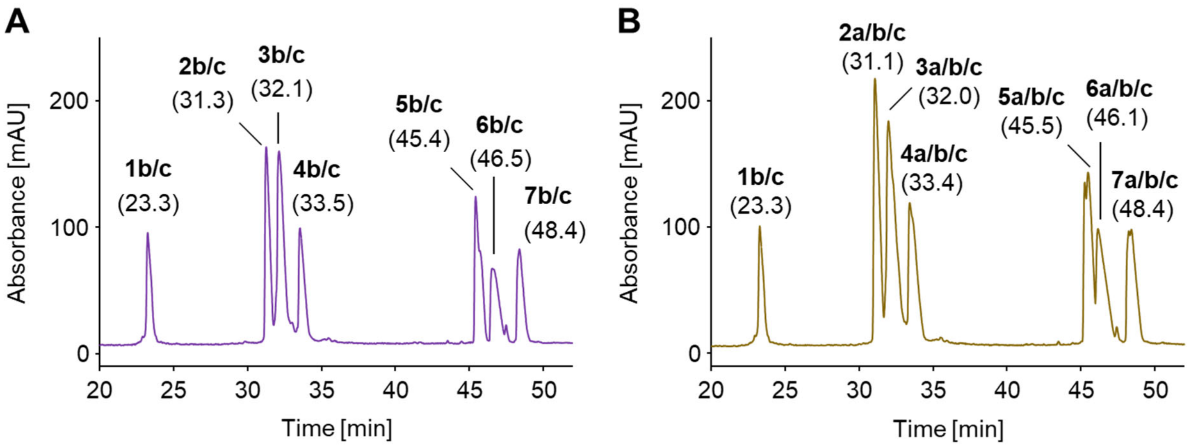

Figure S15: RP chromatograms of mixtures containing glucated and fructated (panel A) or unmodified, glucated and fructated peptides (panel B; 500 pmol each) displayed from 20 to 52 min. Peptides were separated by RP-HPLC (Jupiter C_{18}-column, 60 °C) using a linear 55-min gradient from 3% to 36% aqueous acetonitrile containing 0.1% formic acid. Absorbance was recorded at 214 nm. Full chromatograms are provided in the Supplement (Fig. S14). Peptide sequences and modification sites are provided in Tab. 1.

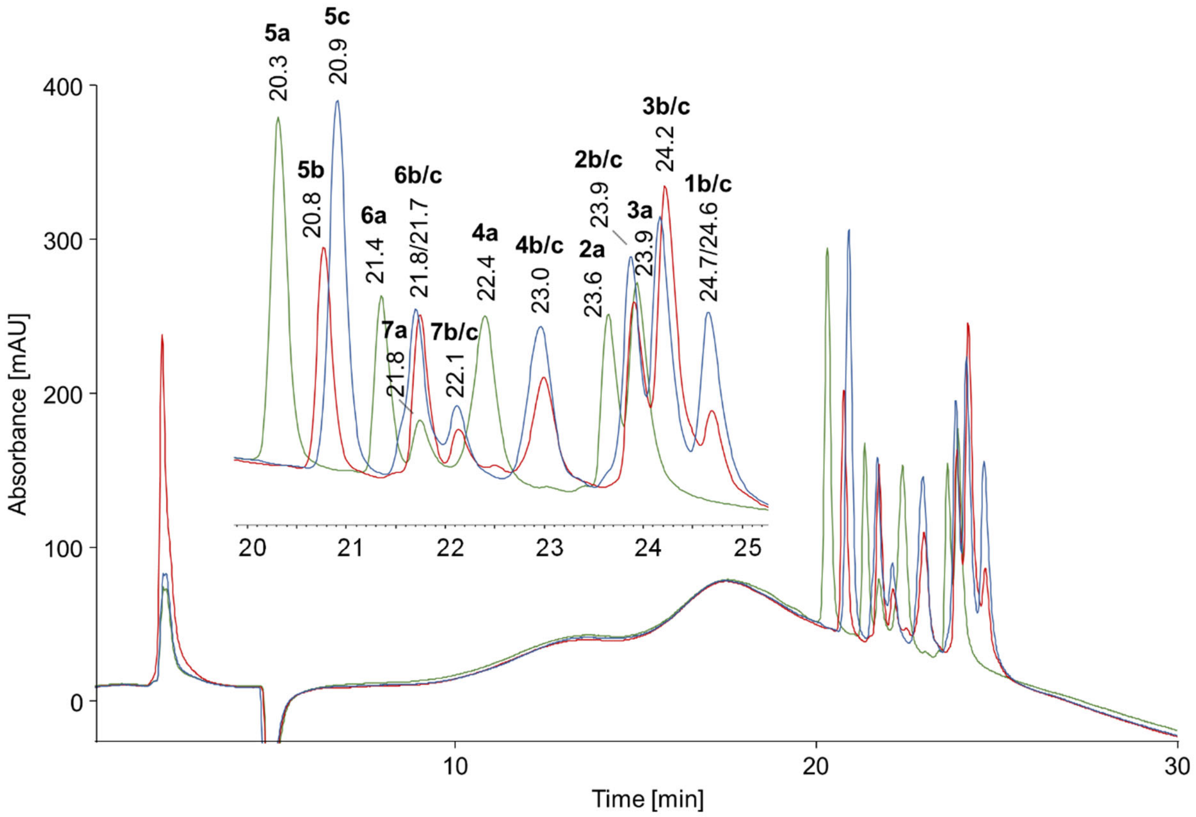

Figure S16: Overlay of HILIC chromatograms of glucated (red trace, n=7), fructated (blue trace, n=7) and unmodified peptides (green trace, n=6). Compounds were analyzed on a Luna HILIC-column (crosslinked diol phase) using a linear 20-min gradient from 88% to 52% aqueous acetonitrile (1.8% H_2O per min) containing ammonium formate (5 mmol/L, pH 3.2). Separations were performed at room temperature and the absorbance was recorded at 214 nm. The small insert displays the zoomed chromatogram from 20 min to 25 min. Peptide sequences and modification sites are provided in Tab. 1.

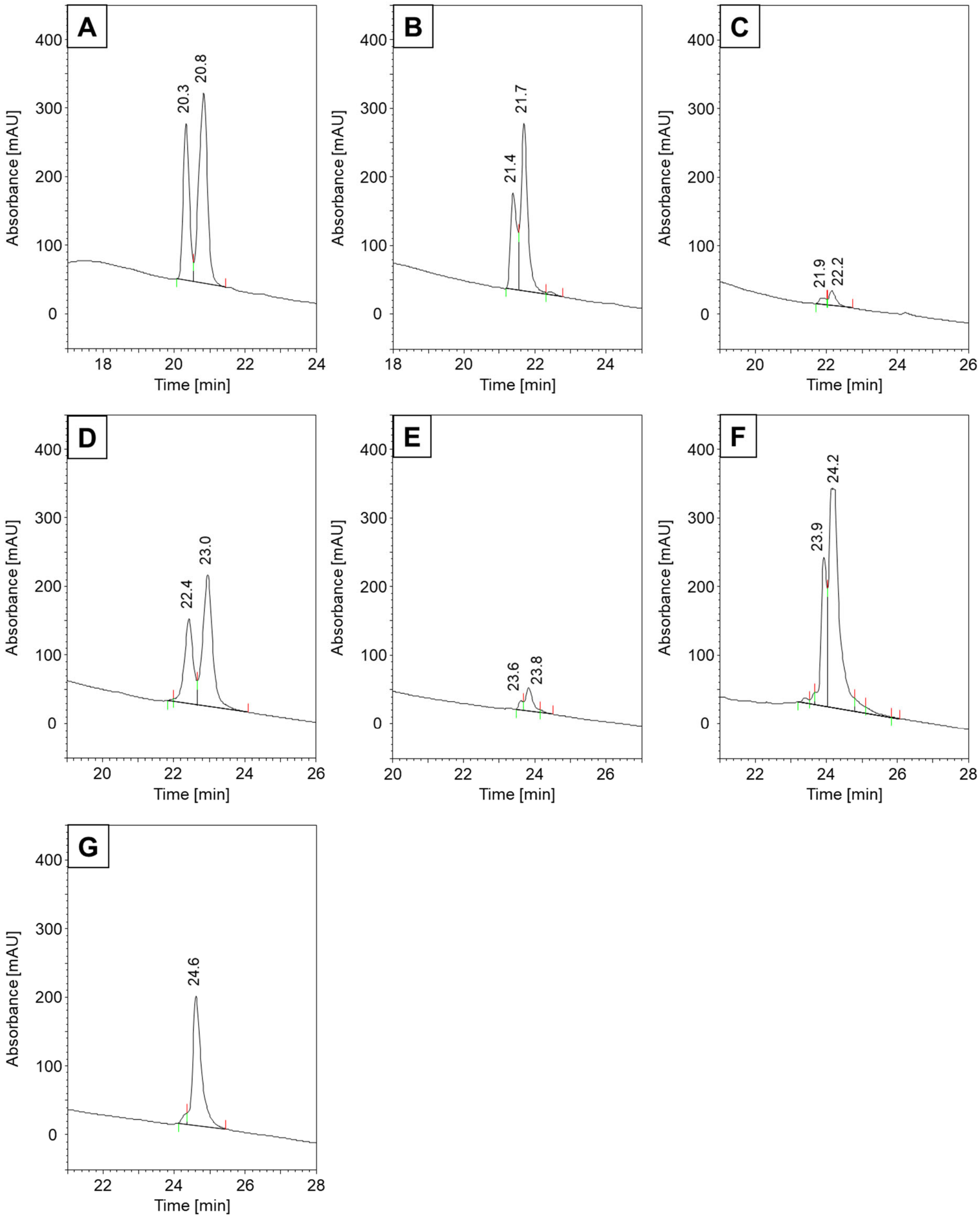

Figure S17: Sections of HILIC chromatograms of peptide families #5 (A), #6 (B), #7 (C), #4 (D), #2 (E), #3 (F), and #1 (G) consisting of glucated, fructated, and unmodified (except #1) peptides (500 pmol each). Peptide mixtures were analyzed on a Luna-HILIC column (room temperature) using a linear 20-min gradient from 88% to 52% aqueous acetonitrile containing ammonium formate (5 mmol/L, pH 3.2). Absorbance was recorded at 214 nm. Peptide sequences and modification sites are provided in Tab. 1.

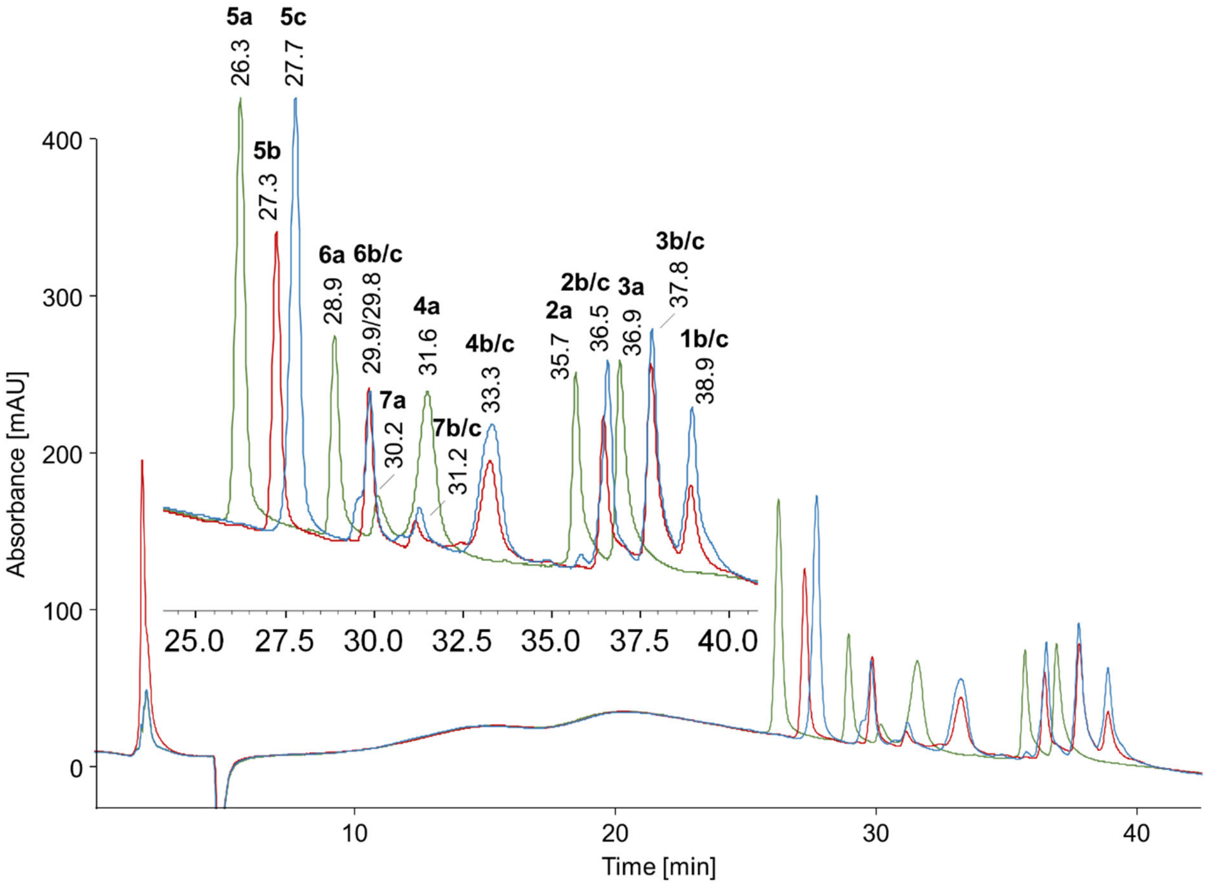

Figure S18: Overlay of HILIC chromatograms of glucated (red trace, n=7), fructated (blue trace, n=7), and unmodified peptides (green trace, n=6). Peptides were separated in HILIC mode on a Luna HILIC-column (crosslinked diol phase) using a linear 60-min gradient from 88% to 52% aqueous acetonitrile (0.6% H_2O per min) containing ammonium formate (5 mmol/L, pH 3.2). Separations were performed at room temperature and the absorbance was recorded at 214 nm. The small insert displays the zoomed chromatogram from 25 min to 40 min. Peptide sequences and modification sites are provided in Tab. 1.
